# Supplementary material for: Cis-regulatory interfaces reveal the molecular mechanisms underlying the notochord gene regulatory network of Ciona
Source: Nat Commun. 2024 Apr 8;15:3025. doi: 10.1038/s41467-024-46850-3 (PMC11001920; doi:10.1038/s41467-024-46850-3)
Supplement: Supplementary file 1 — Supplementary Information [file 41467_2024_46850_MOESM1_ESM.pdf]

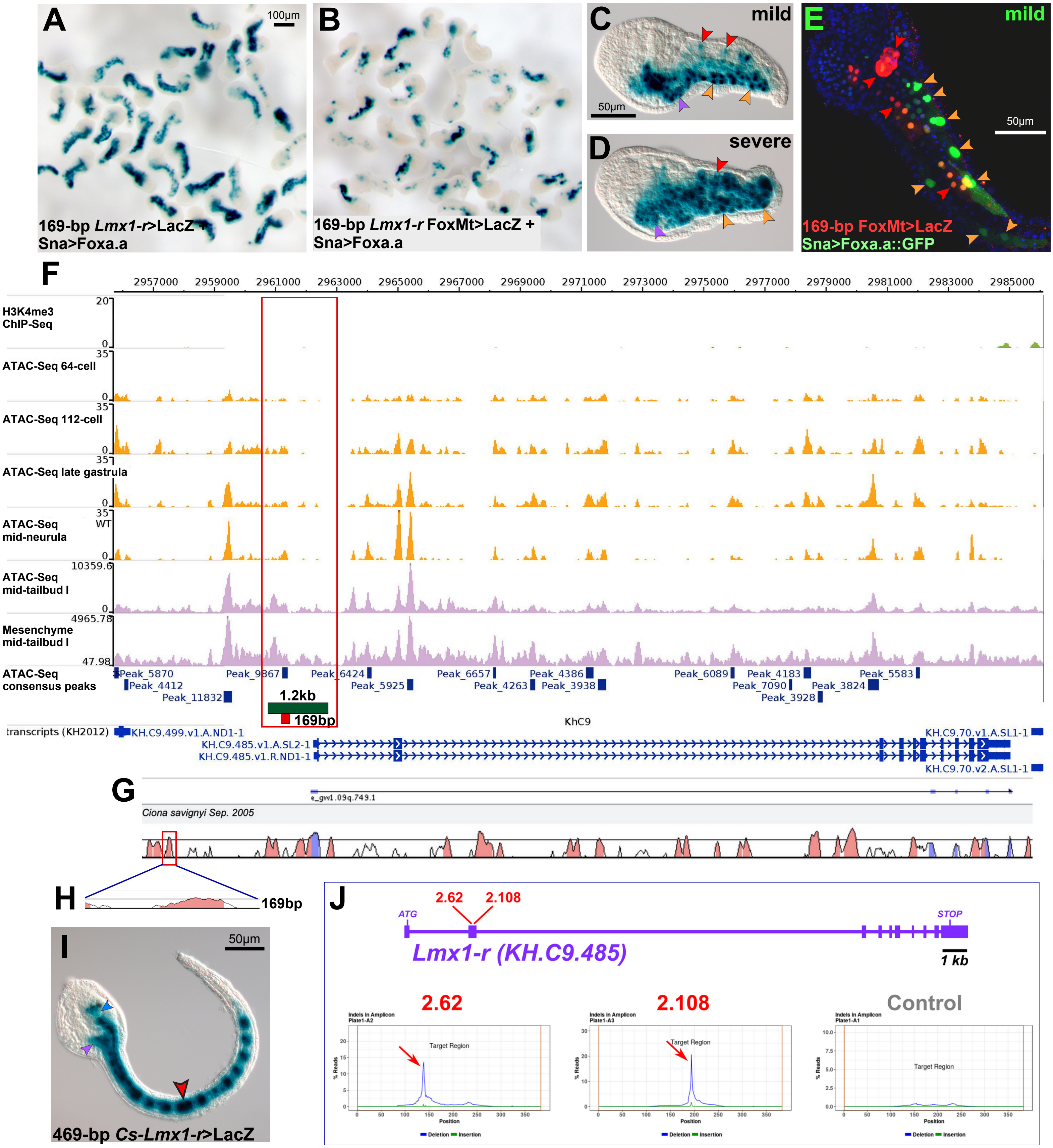

A
100μm
169-bp Lmx1-r>LacZ +
Sna>Foxa.a
B
169-bp Lmx1-r FoxMt>LacZ +
Sna>Foxa.a
C
mild
50μm
D
severe
E
mild
50μm
169-bp FoxMt>LacZ
Sna>Foxa.a::GFP
F
H3K4me3
ChIP-Seq
ATAC-Seq 64-cell
ATAC-Seq 112-cell
ATAC-Seq late gastrula
ATAC-Seq
mid-neurula
ATAC-Seq
mid-tailbud I
Mesenchyme
mid-tailbud I
ATAC-Seq
consensus peaks
1.2kb
169bp
KhC9
transcripts (KH2012)
KH.C9.499.v1.A.ND1-1
KH.C9.485.v1.A.SL2-1
KH.C9.485.v1.R.ND1-1
KH.C9.70.v1.A.SL1-1
KH.C9.70.v2.A.SL1-1
G
Ciona savignyi Sep. 2005
H
169bp
I
50μm
469-bp Cs-Lmx1-r>LacZ
J
2.62
2.108
ATG
STOP
Lmx1-r (KH.C9.485)
1 kb
2.62
2.108
Control
Target Region
Position
Deletion
Insertion

Figure S1. Response of the 169-bp *Lmx1-r* notochord enhancer to the misexpression of Foxa.a in muscle cells; chromatin accessibility and interspecific conservation of the *Lmx1-r* genomic locus; test of the *Lmx1-r* sgRNAs efficiency.

(A,B) Low-magnification group microphotographs of *C. robusta* mid-tailbud embryos electroporated with the transgenes indicated in the lower left corner of each panel. (C,D) High-magnification microphotographs of representative embryos from the experiments in (A,B), showing a 'mild' phenotype (tail partly extended and some structures still distinguishable) (C), and a 'severe' phenotype (stumpy tail, disorganized notochord) (D). (E) Merged confocal microphotograph of a mid-tailbud embryo carrying the transgenes indicated in the bottom left corner, immunostained for beta-galactosidase. Milder phenotype, anterior on top; compare to Fig. 1N and to (C,D) in this figure. (F) Map of the active histone mark H3K4me3 ChIP-Seq plots (gastrula stage)[134] and chromatin accessibility landscape of the *Lmx1-r* genomic locus, determined by ATAC-Seq experiments at the stages indicated on the left side[52,53]. Green horizontal bar: the longest fragment with notochord activity identified in this study (1.2 kb); red rectangle: the 169-bp notochord CRM used for the mutation analysis. Screenshot in (F) reproduced with permission from Aniseed[132] (https://www.aniseed.cnrs.fr). (G) VISTA[64] plot of the sequence conservation between *C. robusta* and *C. savignyi*, reproduced with permission (https://genome.lbl.gov/vista/index.shtml). Conserved non-coding regions are colored in pink, conserved coding regions in blue. (H) Close-up showing the conservation profile of the 169-bp *Lmx1-r* notochord CRM. Interspecific alignment of the binding site necessary for activity is reported in Table S3. (I) *C. robusta* late tailbud embryo carrying a *C. savignyi* genomic fragment encompassing the region in (H). Arrowheads are color-coded

as in Fig. 1; orange arrowheads: muscle staining. (J) Top: The *Lmx-1r* locus, with exons represented by thicker rectangles. sgRNA 2.62 and 2.108 targets in exon 2 are indicated. Bottom: Indel analysis plot from next-generation sequencing of amplicons from larvae electroporated with each sgRNA or with a negative control sgRNA, obtained using the Azenta E-Z amplicon service (see Methods). Red arrows indicate CRISPR/Cas9-induced indel peaks. Note the different scale of y-axis for each plot. Automated analysis of indel plots by Azenta yielded efficacies of ~19% and ~28% for sgRNAs 2.62 and 2.108, respectively.

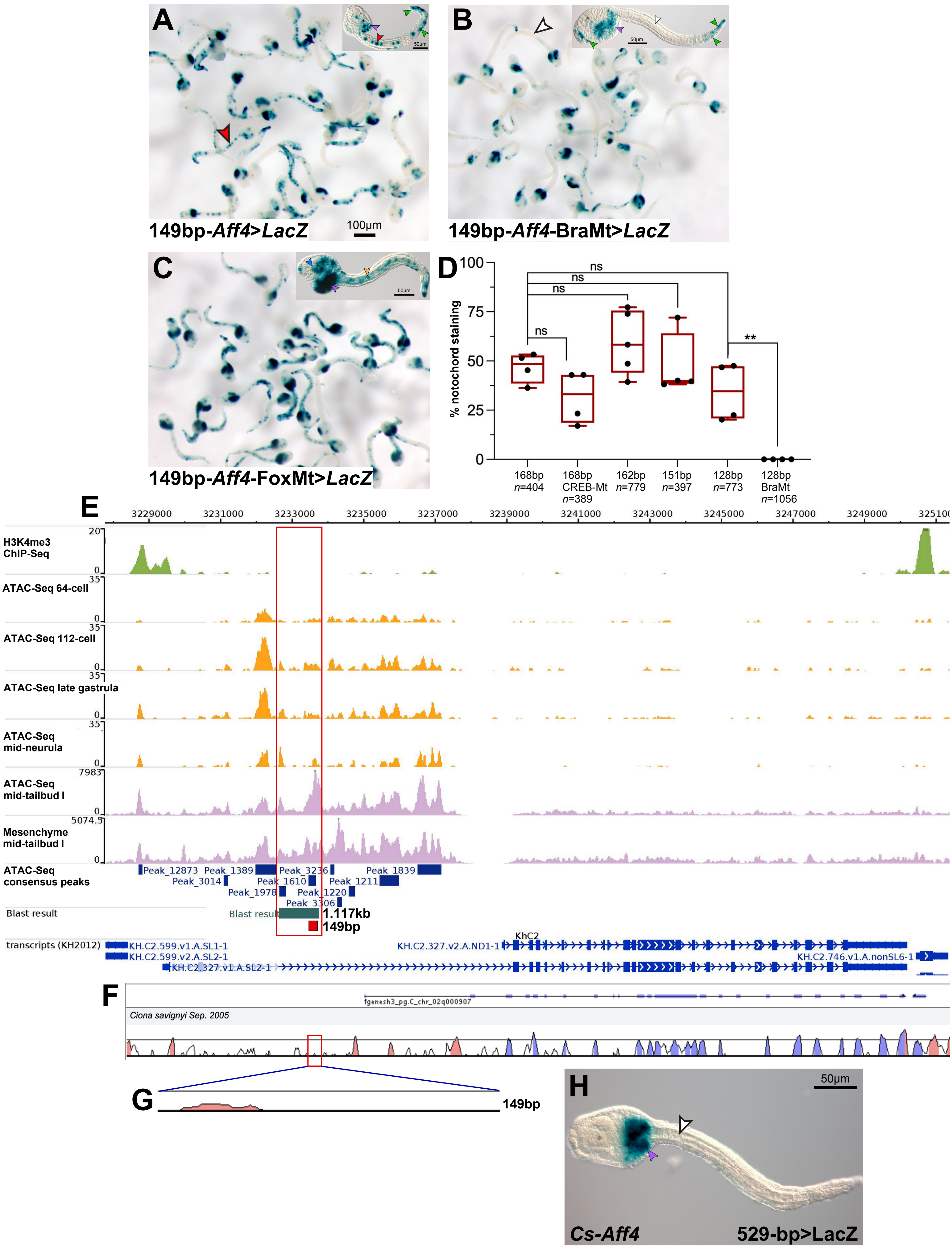
A
149bp-Aff4>LacZ
100μm
50μm
B
149bp-Aff4-BraMt>LacZ
C
149bp-Aff4-FoxMt>LacZ
D
% notochord staining
ns
**
168bp n=404
168bp CREB-Mt n=389
162bp n=779
151bp n=397
128bp n=773
128bp BraMt n=1056
E
H3K4me3 ChIP-Seq
ATAC-Seq 64-cell
ATAC-Seq 112-cell
ATAC-Seq late gastrula
ATAC-Seq mid-neurula
ATAC-Seq mid-tailbud I
Mesenchyme mid-tailbud I
ATAC-Seq consensus peaks
Blast result
transcripts (KH2012)
1.117kb
149bp
KhC2
F
Ciona savignyi Sep. 2005
G
149bp
H
Cs-Aff4
529-bp>LacZ

Figure S2. Mutation analysis of the *Aff4* notochord CRM and overview of chromatin accessibility and interspecific conservation of the *Aff4* genomic locus.

(A-C) Low-magnification group microphotographs of *C. robusta* mid-tailbud embryos electroporated at the 1-cell stage with the transgenes indicated in the lower left corner of each panel, fixed and X-gal stained. (Insets in A-C) High-magnification microphotographs of representative embryos from each experiment. Arrowheads are color-coded as in Fig. 1. (D) Quantification of the results of the truncation/mutation analyses. Each bar is the result of 3-7 biological replicates (black dots). The total number of stained embryos (*n*) analyzed per experiment is shown underneath the *x*-axis. In the graph, two-sided *t*-test significance, whiskers, and other features are as in Fig. 1. Source data are provided as a Source Data file. (E) Map of the active histone mark H3K4me3 ChIP-Seq plots (gastrula stage)[134] and chromatin accessibility landscape of the *Aff4* genomic locus, determined by ATAC-Seq experiments at the stages indicated on the left side[52,53]. Green horizontal bar: the longest fragment with notochord activity identified in this study (1.117 kb); red rectangle: the 149-bp notochord CRM used for the mutation analysis. Blue gene models are from the Kyoto-Hoya (KH) gene assembly[135]; newest gene models (KY gene models[19]) are reported in Table 1. Screenshot in (E) reproduced with permission from Aniseed[132]. (F) VISTA[64] plot of the sequence conservation between *C. robusta* and *C. savignyi*, reproduced with permission. Conserved non-coding regions are colored in pink, conserved coding regions in blue. (G) Close-up showing the conservation profile of the 149-bp *Aff4* notochord CRM. Interspecific alignment of the binding site necessary for activity is reported in

Table S3. (H) *C. robusta* late tailbud embryo carrying a *C. savignyi* genomic fragment encompassing the region in (G).

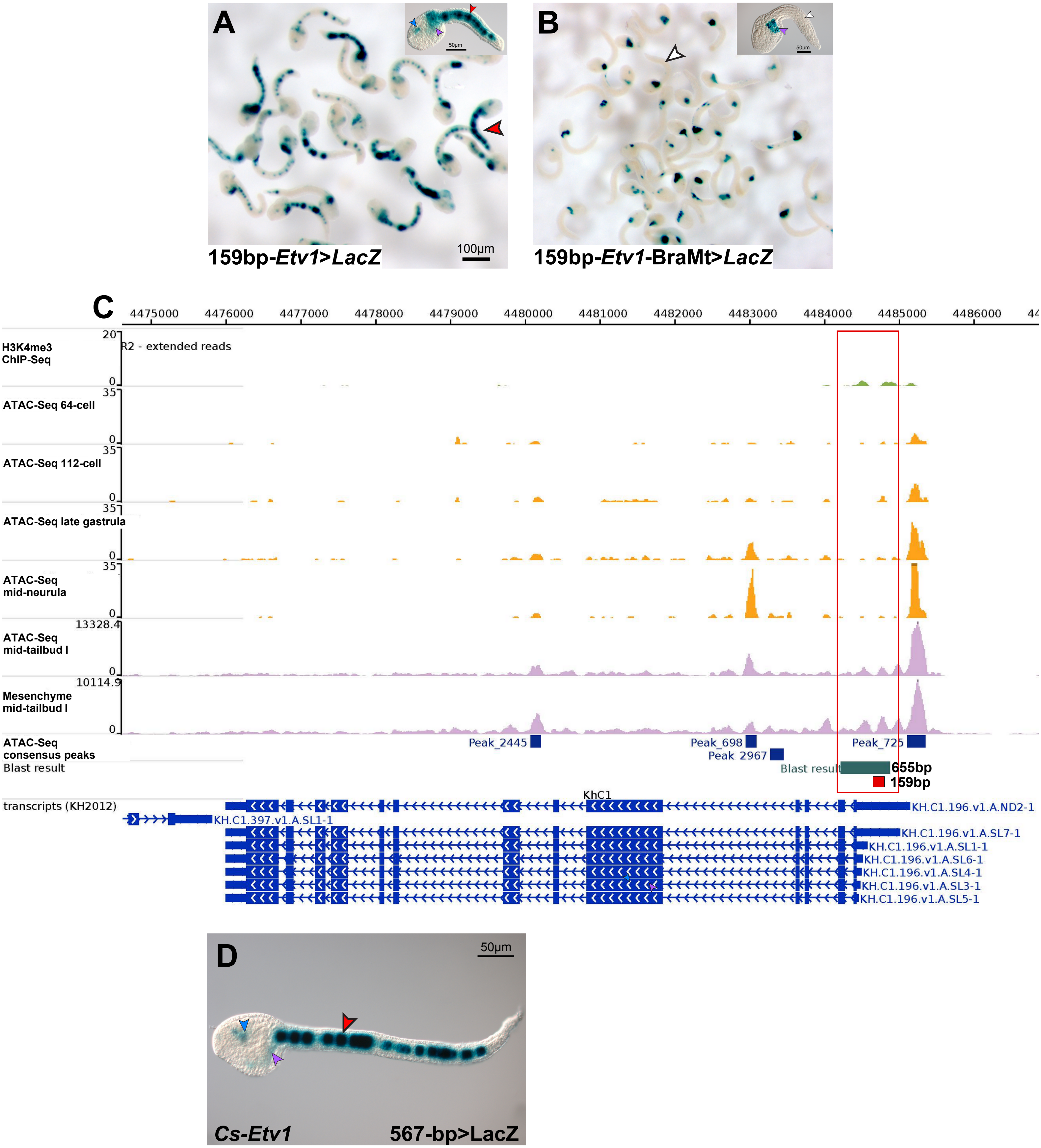
A
159bp-Etv1>LacZ
100µm
50µm
B
159bp-Etv1-BraMt>LacZ
50µm
C
4475000
4476000
4477000
4478000
4479000
4480000
4481000
4482000
4483000
4484000
4485000
4486000
H3K4me3 ChIP-Seq
R2 - extended reads
ATAC-Seq 64-cell
ATAC-Seq 112-cell
ATAC-Seq late gastrula
ATAC-Seq mid-neurula
ATAC-Seq mid-tailbud I
13328.4
Mesenchyme mid-tailbud I
10114.9
ATAC-Seq consensus peaks
Peak_2445
Peak_698
Peak_2967
Peak_725
Blast result
655bp
159bp
transcripts (KH2012)
KhC1
KH.C1.196.v1.A.ND2-1
KH.C1.397.v1.A.SL1-1
KH.C1.196.v1.A.SL7-1
KH.C1.196.v1.A.SL1-1
KH.C1.196.v1.A.SL6-1
KH.C1.196.v1.A.SL4-1
KH.C1.196.v1.A.SL3-1
KH.C1.196.v1.A.SL5-1
D
50µm
Cs-Etv1
567-bp>LacZ

Figure S3. Mutation analysis of the *Etv1* notochord CRM and overview of chromatin accessibility and interspecific conservation of the *Etv1* genomic locus.

(A,B) Low-magnification group microphotographs of *C. robusta* mid-tailbud embryos electroporated with the transgenes indicated in the lower left corner of each panel, fixed and stained for beta-galactosidase. Insets: High-magnification microphotographs of representative embryos from each experiment. Arrowheads are color-coded as in Fig. 1. (C) Map of the active histone mark H3K4me3 ChIP-Seq plots (gastrula stage)[134] and chromatin accessibility of the *Etv1* genomic locus, determined by ATAC-Seq[52,53]. Green horizontal bar: the longest fragment with notochord activity identified in this study (655 bp); red rectangle: the 159-bp notochord CRM. Blue gene models are from the Kyoto-Hoya (KH) gene assembly[135]; newest gene models (KY gene models[19]) are reported in Table 1. Screenshot in (C) reproduced with permission from Aniseed[132]. VISTA comparisons did not identify sequence alignment in the region harboring the notochord CRM, likely due to gaps in the genome assemblies used by this computational tool; interspecific conservation of the binding site necessary for activity is reported in Table S3. (D) *C. robusta* late tailbud embryo carrying a *C. savignyi* genomic fragment corresponding to the *Etv1* notochord CRM (Fig. 3).

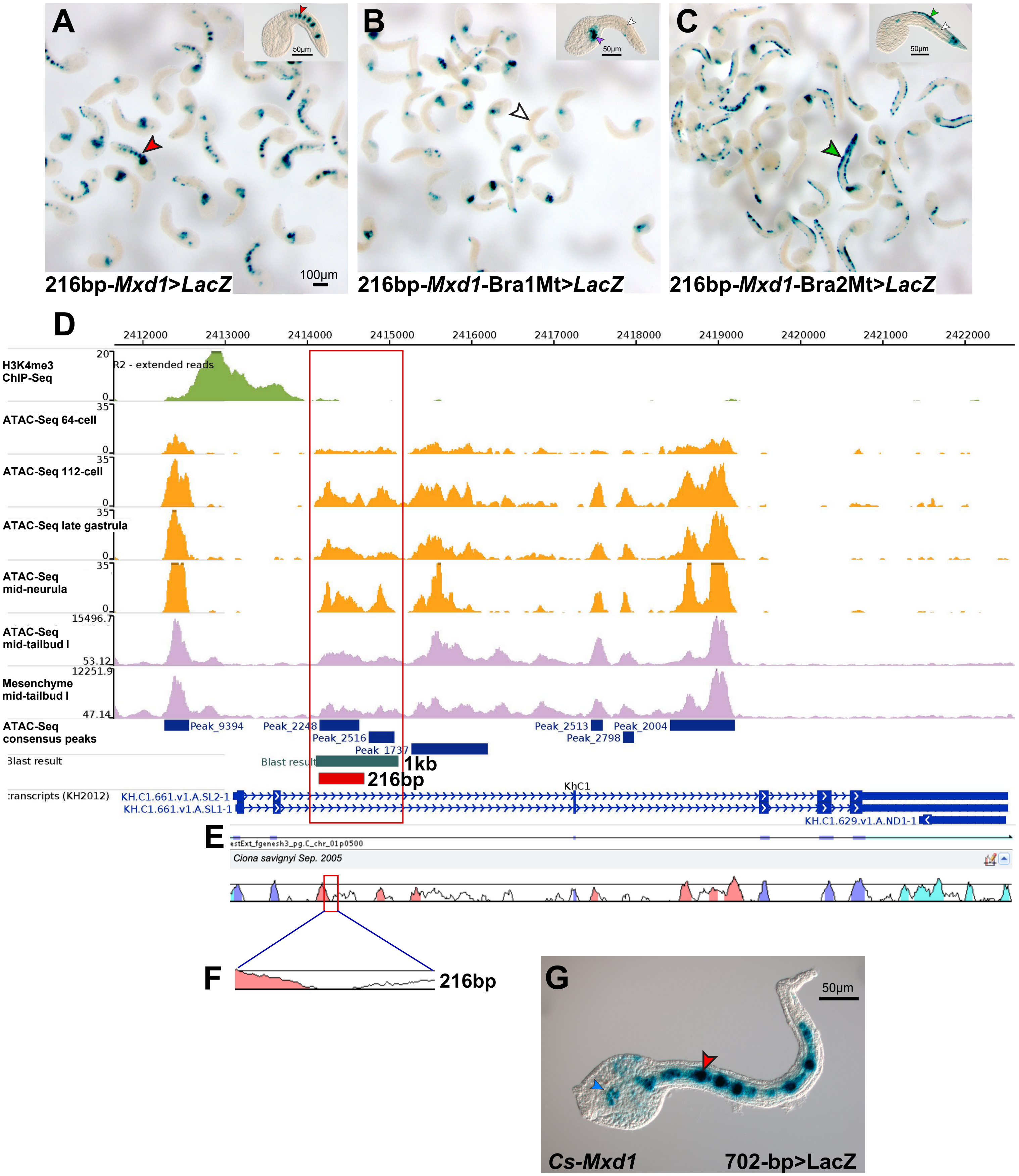

A
50µm
100µm
216bp-Mxd1>LacZ
B
50µm
216bp-Mxd1-Bra1Mt>LacZ
C
50µm
216bp-Mxd1-Bra2Mt>LacZ
D
2412000
2413000
2414000
2415000
2416000
2417000
2418000
2419000
2420000
2421000
2422000
H3K4me3 ChIP-Seq
R2 - extended reads
ATAC-Seq 64-cell
ATAC-Seq 112-cell
ATAC-Seq late gastrula
ATAC-Seq mid-neurula
ATAC-Seq mid-tailbud I
Mesenchyme mid-tailbud I
ATAC-Seq consensus peaks
Peak_9394
Peak_2248
Peak_2516
Peak_1737
Peak_2513
Peak_2798
Peak_2004
Blast result
1kb
216bp
transcripts (KH2012)
KH.C1.661.v1.A.SL2-1
KH.C1.661.v1.A.SL1-1
KhC1
KH.C1.629.v1.A.ND1-1
E
estExt_fgenesh3_pg.C_chr_01p0500
Ciona savignyi Sep. 2005
F
216bp
G
50µm
Cs-Mxd1
702-bp>LacZ

Figure S4. Mutation analysis of the *Mxd1* notochord CRM and overview of chromatin accessibility and interspecific conservation of the *Mxd1* genomic locus.

(A-C) Low-magnification group microphotographs of *C. robusta* mid-tailbud embryos electroporated with the transgenes indicated in the lower left corner of each panel, fixed and stained for beta-galactosidase. (Insets in A-C) High-magnification microphotographs of representative embryos from each experiment. Arrowheads are color-coded as in Fig. 1. (D) Map of the active histone mark H3K4me3 ChIP-Seq plots (gastrula stage)[134] and chromatin accessibility of the *Mxd1* genomic locus, determined by ATAC-Seq experiments[52,53]. Green horizontal bar: the longest fragment with notochord activity identified in this study (1 kb); red rectangle: the 216-bp notochord CRM used for the mutation analysis. Blue gene models are from the Kyoto-Hoya (KH) gene assembly[135]; newest gene models (KY gene models[19]) are reported in Table 1. Screenshot in (D) reproduced with permission from Aniseed[132]. (E) VISTA[64] plot of the sequence conservation between *C. robusta* and *C. savignyi*, reproduced with permission. Conserved non-coding regions are colored in pink, conserved coding regions in blue, 5'- and 3'-untranslated regions in aqua. (F) Close-up of the conservation profile of the 216-bp *Mxd1* notochord CRM. Interspecific conservation of the binding site necessary for activity is reported in Table S3. (G) *C. robusta* late tailbud embryo carrying a *C. savignyi* genomic fragment encompassing the region in (G).

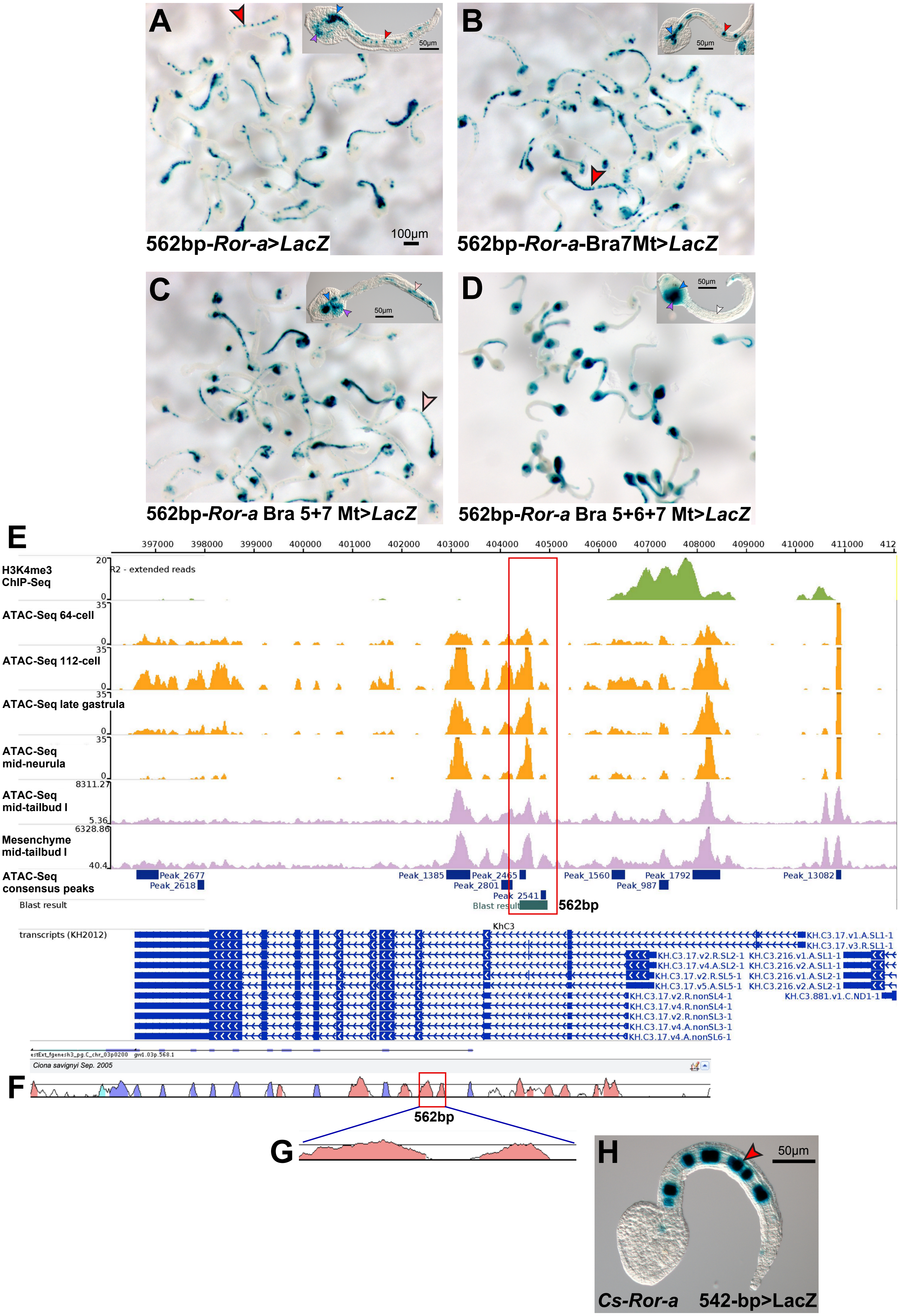

A
50µm
100µm
562bp-Ror-a>LacZ
B
50µm
562bp-Ror-a-Bra7Mt>LacZ
C
50µm
562bp-Ror-a Bra 5+7 Mt>LacZ
D
50µm
562bp-Ror-a Bra 5+6+7 Mt>LacZ
E
H3K4me3 ChIP-Seq
ATAC-Seq 64-cell
ATAC-Seq 112-cell
ATAC-Seq late gastrula
ATAC-Seq mid-neurula
ATAC-Seq mid-tailbud I
Mesenchyme mid-tailbud I
ATAC-Seq consensus peaks
Blast result
transcripts (KH2012)
Peak_2677
Peak_2618
Peak_1385
Peak_2465
Peak_2801
Peak_2541
Peak_1560
Peak_987
Peak_1792
Peak_13082
562bp
KhC3
Ciona savignyi Sep. 2005
F
562bp
G
H
50µm
Cs-Ror-a 542-bp>LacZ

Figure S5. Mutation analysis of the *Ror-a* notochord CRM and overview of chromatin accessibility and interspecific conservation of the *Ror-a* genomic locus.

(A-D) Low-magnification group microphotographs of *C. robusta* mid-tailbud embryos electroporated with the transgenes indicated in the lower left corner of each panel, fixed and stained for beta-galactosidase. Insets in (A-D): High-magnification microphotographs of representative embryos from each experiment. Arrowheads are color-coded as in Fig. 1. (E) Map of the active histone mark H3K4me3 ChIP-Seq plots (gastrula stage)[134] and chromatin accessibility landscape of the *Ror-a* genomic locus, determined by ATAC-Seq experiments[52,53]. Green horizontal bar: the 562-bp notochord enhancer region, used for mutation analysis. Blue gene models are from the Kyoto-Hoya (KH) gene assembly[135]; newest gene models (KY gene models[19]) are reported in Table 1. Screenshot in (E) reproduced with permission from Aniseed[132]. (F) VISTA[64] plot of the sequence conservation between *C. robusta* and *C. savignyi*, reproduced with permission. Conserved non-coding regions are colored in pink, conserved coding regions in blue, 5'- and 3'-untranslated regions in aqua. (G) Conservation profile of the 562-bp *Ror-a* notochord enhancer region. (H) *C. robusta* late tailbud embryo carrying a *C. savignyi* genomic fragment corresponding to the *Ror-a* notochord CRM (Fig. 3).

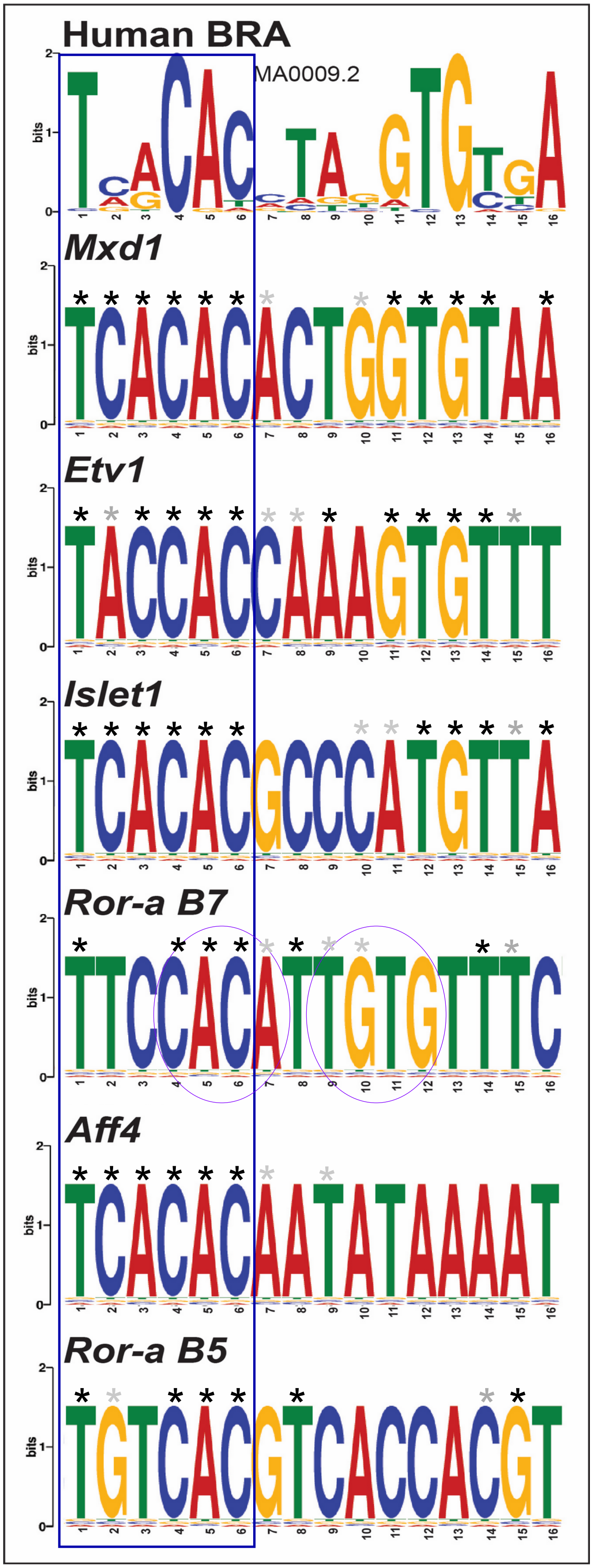

Human BRA
MA0009.2
Mxd1
Etv1
Islet1
Ror-a B7
Aff4
Ror-a B5

Figure S6. Sequence and structure of the functional T-box binding sites identified in this study, compared to the consensus binding site for human BRACHYURY.

Palindromic consensus binding site for human BRA. Two of the functional Bra/T-box binding sites identified in this study, *Mxd1* and *Etv1* (Fig. 3) show the same palindromic structure and spacing seen in the human BRA consensus binding site. B7, one of the two Bra/T-box binding sites necessary for the activity of the *Ror-a* notochord CRM (Fig. 3V), has a partial palindromic organization (ovals), although the spacing between its two half-sites is reduced (from 4 bp to 3 bp) compared to the human consensus. Of the remaining Bra/T-box binding sites, *Islet1* (Fig. 5) has a less stringent palindromic structure, while the Bra/T-box binding site in the *Aff4* notochord CRM and the B5 Bra/T-box binding site necessary for the activity of the *Ror-a* notochord CRM (Fig. 3V) are structured as TNNCAC half-sites (framed in blue). Black/grey asterisks mark conservation between the *Ciona* Bra/T-box binding sites and the human BRA consensus.

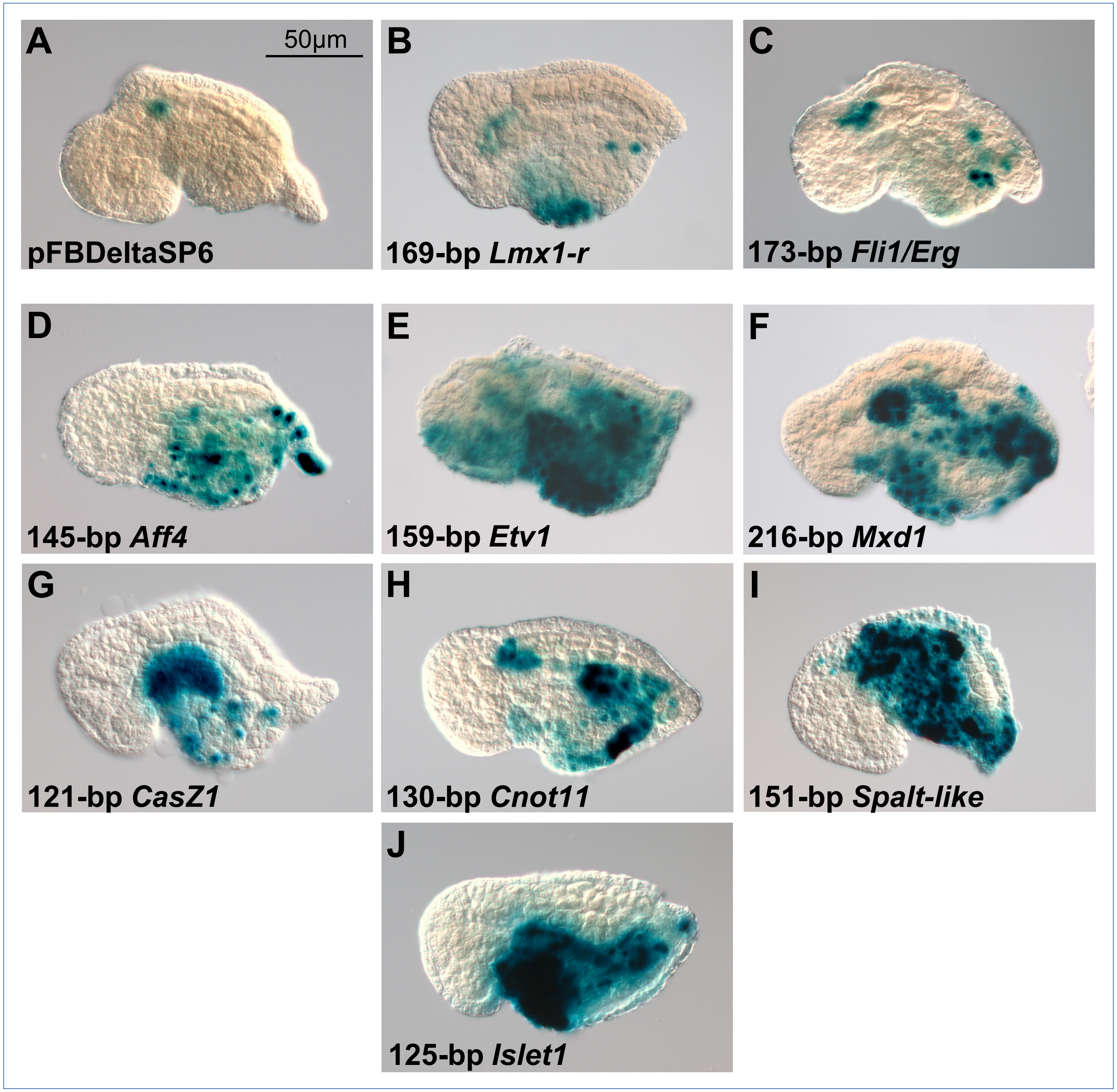
A
50μm
pFBDeltaSP6
B
169-bp *Lmx1-r*
C
173-bp *Fli1/Erg*
D
145-bp *Aff4*
E
159-bp *Etv1*
F
216-bp *Mxd1*
G
121-bp *CasZ1*
H
130-bp *Cnot11*
I
151-bp *Spalt-like*
J
125-bp *Islet1*

Figure S7. ***Trans*****-activation assays shed light on branches of the notochord GRN controlled indirectly by Ci-Bra.**

Mid/late-tailbud X-gal-stained *C. robusta* embryos carrying the *Foxa.a>Bra* plasmid, which ectopically expresses Ci-Bra in CNS and endoderm, and the *LacZ*-reporter plasmids indicated in the lower left corner of each panel. (A) Control embryo electroporated with the vector pFBΔSP6[56], which is not responsive to the ectopic expression of Ci-Bra. (B,C) Embryos carrying notochord CRMs that do not respond to the ectopic expression of Ci-Bra. The co-electroporations of the vector and of the *Lmx1-r* and *Etv1* notochord CRMs with *Foxa.a>Bra* are also shown in Fig. 4, and are repeated here to provide a reference for the extent and levels of X-gal staining. (D-J) Embryos carrying notochord CRMs that respond to the ectopic expression of Ci-Bra. Control embryos for these experiments are shown in Figs. 1, 2 and 4.

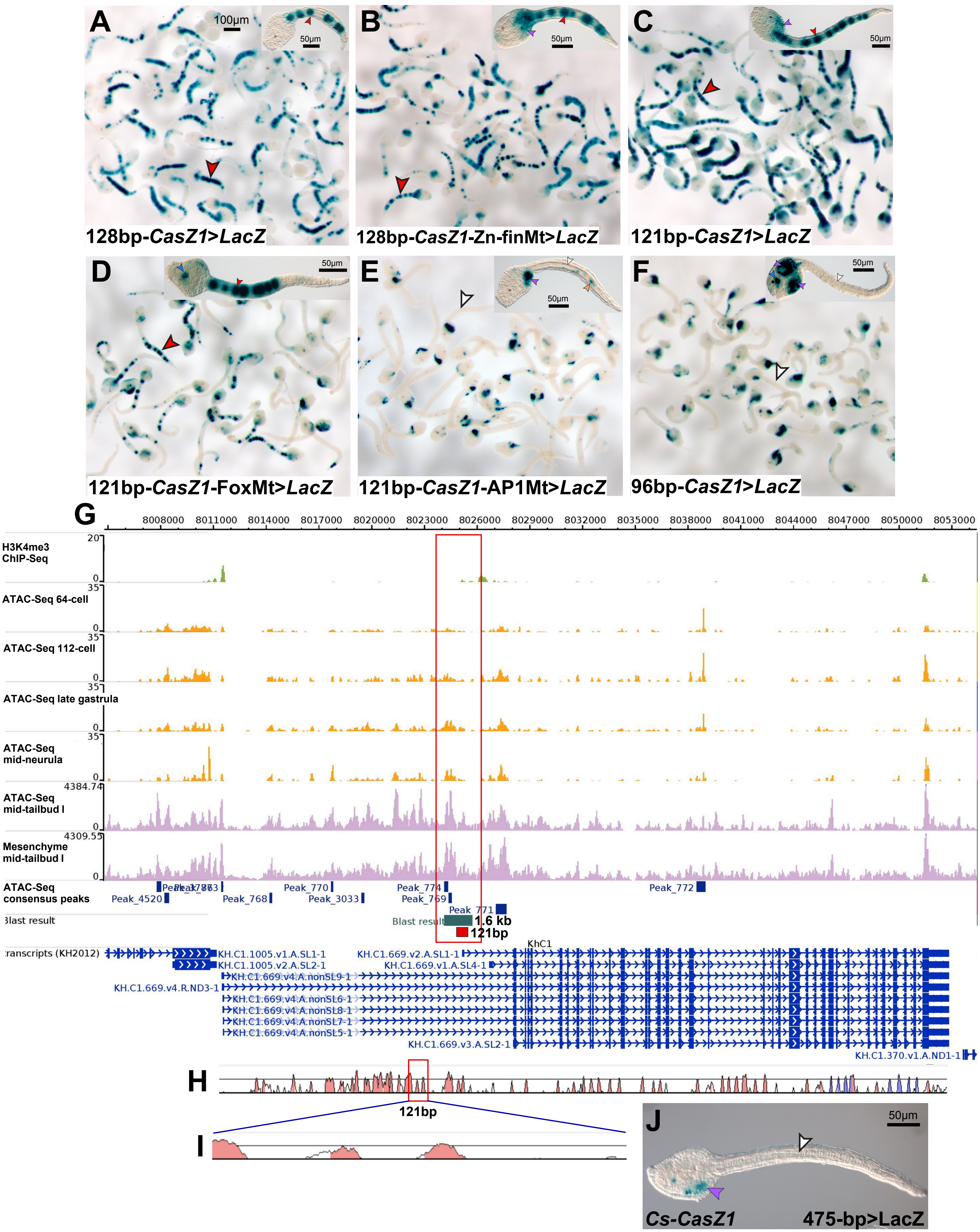

A
100µm
50µm
128bp-CasZ1>LacZ
B
50µm
128bp-CasZ1-Zn-finMt>LacZ
C
50µm
121bp-CasZ1>LacZ
D
50µm
121bp-CasZ1-FoxMt>LacZ
E
50µm
121bp-CasZ1-AP1Mt>LacZ
F
50µm
96bp-CasZ1>LacZ
G
8008000 8011000 8014000 8017000 8020000 8023000 8026000 8029000 8032000 8035000 8038000 8041000 8044000 8047000 8050000 8053000
H3K4me3 ChIP-Seq
ATAC-Seq 64-cell
ATAC-Seq 112-cell
ATAC-Seq late gastrula
ATAC-Seq mid-neurula
ATAC-Seq mid-tailbud I
Mesenchyme mid-tailbud I
ATAC-Seq consensus peaks
Peak_4520
Peak_768
Peak_770
Peak_3033
Peak_774
Peak_769
Peak_771
Peak_772
Blast result
1.6 kb
121bp
KhC1
KH.C1.1005.v1.A.SL1-1
KH.C1.1005.v2.A.SL2-1
KH.C1.669.v2.A.SL1-1
KH.C1.669.v1.A.SL4-1
KH.C1.669.v4.A.nonSL9-1
KH.C1.669.v4.R.ND3-1
KH.C1.669.v4.A.nonSL6-1
KH.C1.669.v4.A.nonSL8-1
KH.C1.669.v4.A.nonSL7-1
KH.C1.669.v4.A.nonSL5-1
KH.C1.669.v3.A.SL2-1
KH.C1.370.v1.A.ND1-1
H
121bp
I
J
50µm
Cs-CasZ1
475-bp>LacZ

Figure S8. Mutation analysis of the *CasZ1* notochord CRM and overview of chromatin accessibility and interspecific conservation of the *CasZ1* genomic locus.

(A-F) Low-magnification group microphotographs of *C. robusta* mid-tailbud embryos electroporated with the transgenes indicated in the lower left corner of each panel, fixed and X-gal stained. (Insets in A-F) High-magnification microphotographs of representative embryos from each experiment. Arrowheads are color-coded as in Fig. 1. (G) Map of the active histone mark H3K4me3 ChIP-Seq plots (gastrula stage)[134] and chromatin accessibility of the *CasZ1* genomic locus, determined by ATAC-Seq experiments[52,53]. Green horizontal bar: the longest fragment with notochord activity identified in this study (1.6 kb); red rectangle: the 121-bp notochord CRM used for the mutation analysis. Blue gene models are from the Kyoto-Hoya (KH) gene assembly[135]; newest gene models (KY gene models[19]) are reported in Table 1. Screenshot in (G) reproduced with permission from Aniseed[132]. (H) VISTA[64] plot of the sequence conservation between *C. robusta* and *C. savignyi*, reproduced with permission. Conserved non-coding regions are colored in pink, conserved coding regions in blue. (I) Close-up showing the conservation profile of the 121-bp *CasZ1* notochord CRM. Interspecific conservation of the binding sites necessary for activity is shown in Table S3. (J) *C. robusta* late tailbud embryo carrying a *C. savignyi* genomic fragment encompassing the region in (I).

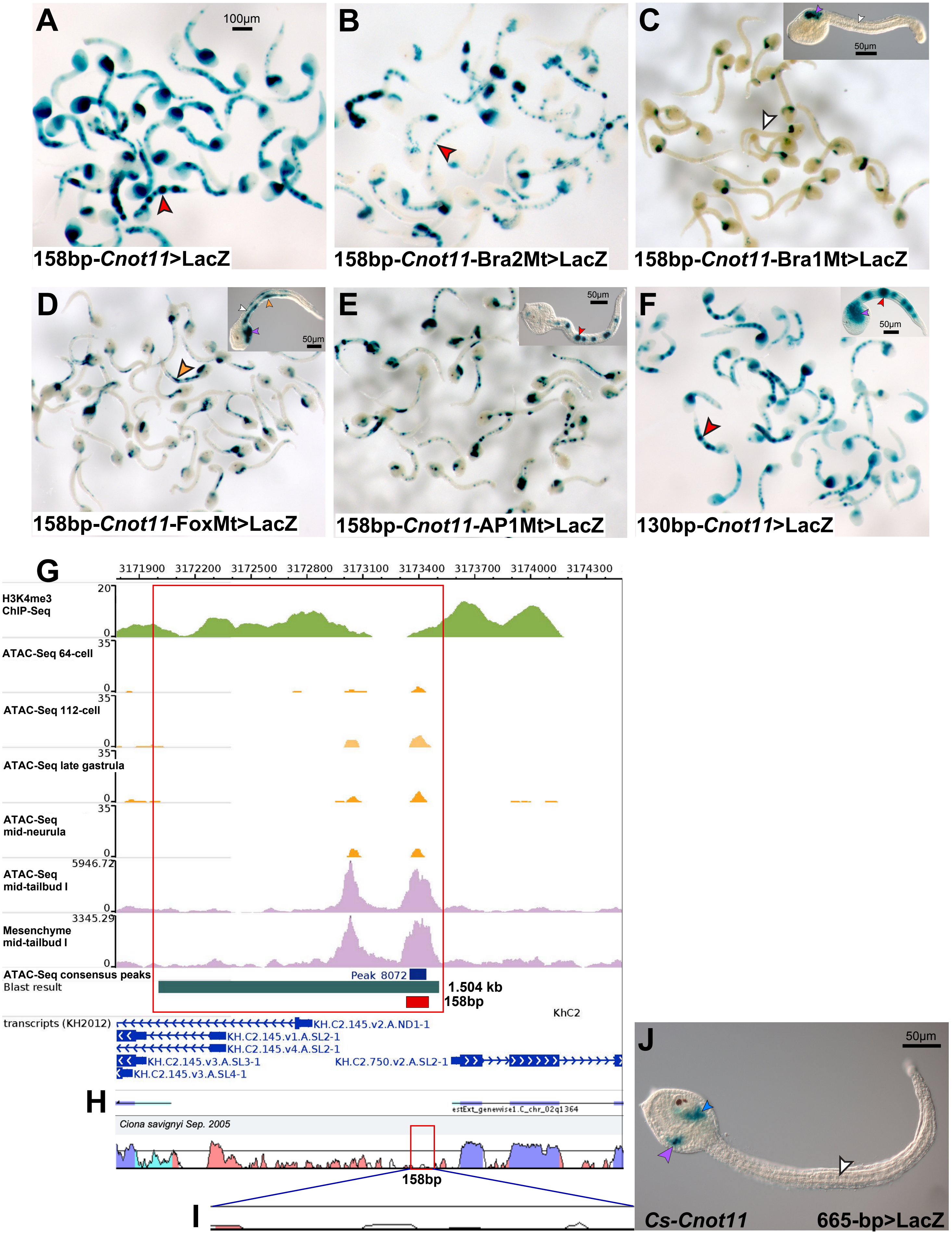

A
100µm
158bp-Cnot11>LacZ
B
158bp-Cnot11-Bra2Mt>LacZ
C
50µm
158bp-Cnot11-Bra1Mt>LacZ
D
50µm
158bp-Cnot11-FoxMt>LacZ
E
50µm
158bp-Cnot11-AP1Mt>LacZ
F
50µm
130bp-Cnot11>LacZ
G
3171900 3172200 3172500 3172800 3173100 3173400 3173700 3174000 3174300
H3K4me3 ChIP-Seq
ATAC-Seq 64-cell
ATAC-Seq 112-cell
ATAC-Seq late gastrula
ATAC-Seq mid-neurula
ATAC-Seq mid-tailbud I
5946.72
Mesenchyme mid-tailbud I
3345.29
ATAC-Seq consensus peaks
Peak 8072
Blast result
1.504 kb
158bp
KhC2
transcripts (KH2012)
KH.C2.145.v2.A.ND1-1
KH.C2.145.v1.A.SL2-1
KH.C2.145.v4.A.SL2-1
KH.C2.145.v3.A.SL3-1
KH.C2.750.v2.A.SL2-1
KH.C2.145.v3.A.SL4-1
H
estExt_genewise1.C_chr_02q1364
Ciona savignyi Sep. 2005
158bp
I
J
50µm
Cs-Cnot11
665-bp>LacZ

Figure S9. Mutation analysis of the *Cnot11* notochord CRM and overview of chromatin accessibility and interspecific conservation of the *Cnot11* genomic locus.

(A-F) Low-magnification group microphotographs of *C. robusta* mid-tailbud embryos electroporated at the 1-cell stage with the transgenes indicated in the lower left corner of each panel, fixed and stained for beta-galactosidase. Insets in (C-F): High-magnification microphotographs of representative embryos from each experiment. Arrowheads are color-coded as in Fig. 1. (G) Map of the active histone mark H3K4me3 ChIP-Seq plots (gastrula stage)[134] and chromatin accessibility landscape of the *Cnot11* genomic locus, determined by ATAC-Seq experiments[52,53]. Green horizontal bar: the longest fragment with notochord activity identified in this study (1.504 kb); red rectangle: the 158-bp notochord CRM used for the mutation analysis. Blue gene models are from the Kyoto-Hoya (KH) gene assembly[135]; newest gene models (KY gene models[19]) are reported in Table 1. Screenshot in (G) reproduced with permission from Aniseed[132]. (H) VISTA[64] plot of the sequence conservation between *C. robusta* and *C. savignyi*, reproduced with permission. Conserved non-coding regions are colored in pink, conserved coding regions in blue, 5'- and 3'-untranslated regions in aqua. (I) Close-up showing the conservation profile of the 158-bp *Cnot11* notochord CRM. (J) *C. robusta* late tailbud embryo carrying a *C. savignyi* genomic fragment encompassing the region in (I).

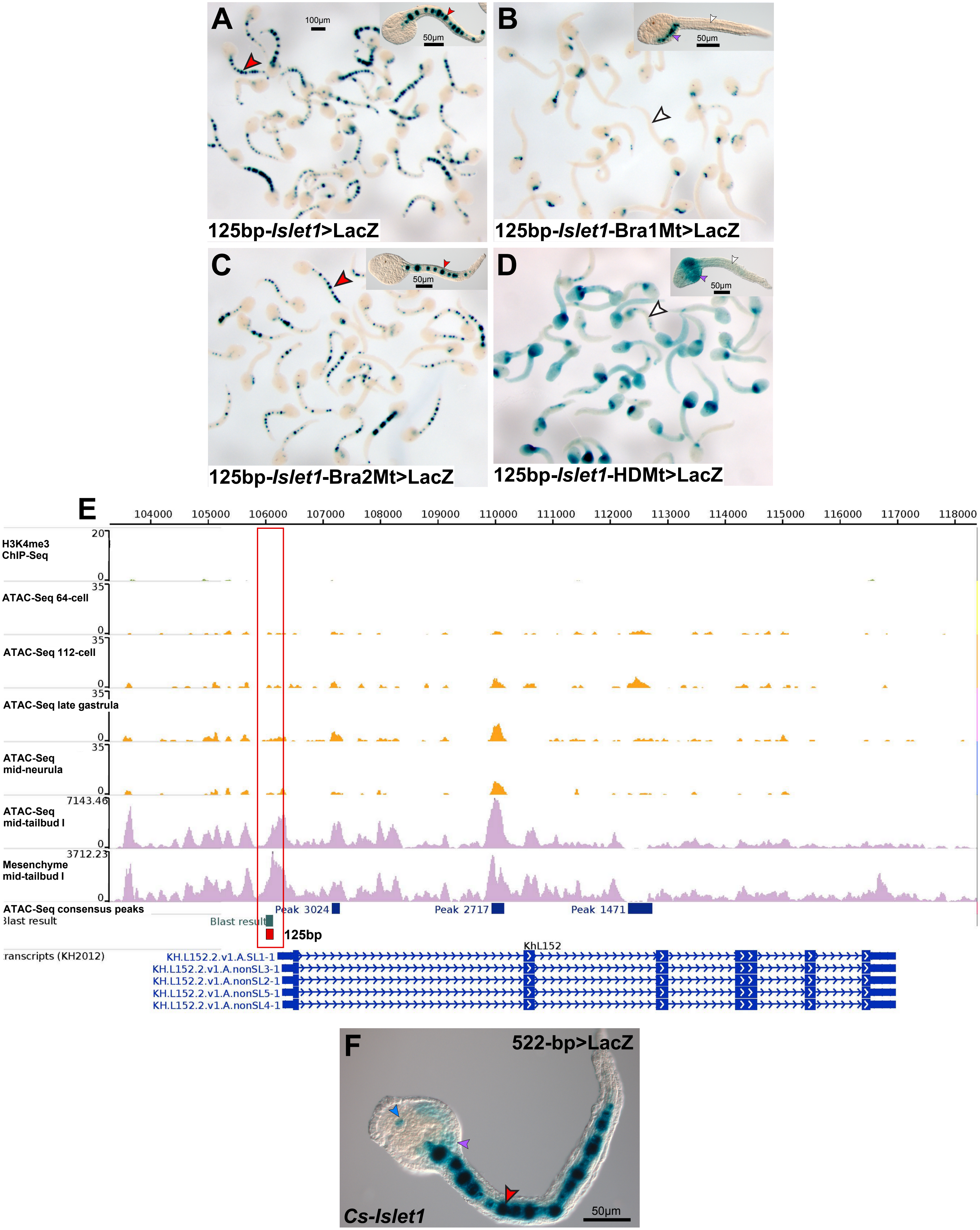
A
100µm
50µm
125bp-Islet1>LacZ
B
50µm
125bp-Islet1-Bra1Mt>LacZ
C
50µm
125bp-Islet1-Bra2Mt>LacZ
D
50µm
125bp-Islet1-HDMt>LacZ
E
104000
105000
106000
107000
108000
109000
110000
111000
112000
113000
114000
115000
116000
117000
118000
H3K4me3 ChIP-Seq
ATAC-Seq 64-cell
ATAC-Seq 112-cell
ATAC-Seq late gastrula
ATAC-Seq mid-neurula
ATAC-Seq mid-tailbud I
Mesenchyme mid-tailbud I
ATAC-Seq consensus peaks
Peak 3024
Peak 2717
Peak 1471
Blast result
125bp
KhL152
KH.L152.2.v1.A.SL1-1
KH.L152.2.v1.A.nonSL3-1
KH.L152.2.v1.A.nonSL2-1
KH.L152.2.v1.A.nonSL5-1
KH.L152.2.v1.A.nonSL4-1
F
522-bp>LacZ
Cs-Islet1
50µm

Figure S10. Mutation analysis of the *Islet1* notochord CRM and overview of chromatin accessibility and interspecific conservation of the *Islet1* genomic locus.

(A-D) Low-magnification group microphotographs of *C. robusta* mid-tailbud embryos electroporated with the transgenes indicated in the lower left corner of each panel, fixed and stained for beta-galactosidase. Insets: high-magnification microphotographs of representative embryos from each experiment. Arrowheads are color-coded as in Fig. 1. (E) Map of the active histone mark H3K4me3 ChIP-Seq plots (gastrula stage)[134] and chromatin accessibility of the *Islet1* genomic locus, determined by ATAC-Seq experiments[52,53]. Green horizontal bar: BLASTN result for the sequence of the 125-bp notochord CRM (red rectangle). Blue gene models are from the Kyoto-Hoya (KH) gene assembly[135]; newest gene models (KY gene models[19]) are reported in Table 1. Screenshot in (E) reproduced with permission from Aniseed[132]. VISTA[64] comparisons did not identify sequence conservation in the region harboring the notochord CRM, likely due to gaps in the genome assemblies used by this computational tool; interspecific conservation of the binding sites necessary for activity is reported in Table S3. (F) *C. robusta* late tailbud embryo carrying a *C. savignyi* genomic fragment corresponding to the *Islet1* notochord CRM (Fig. 5).

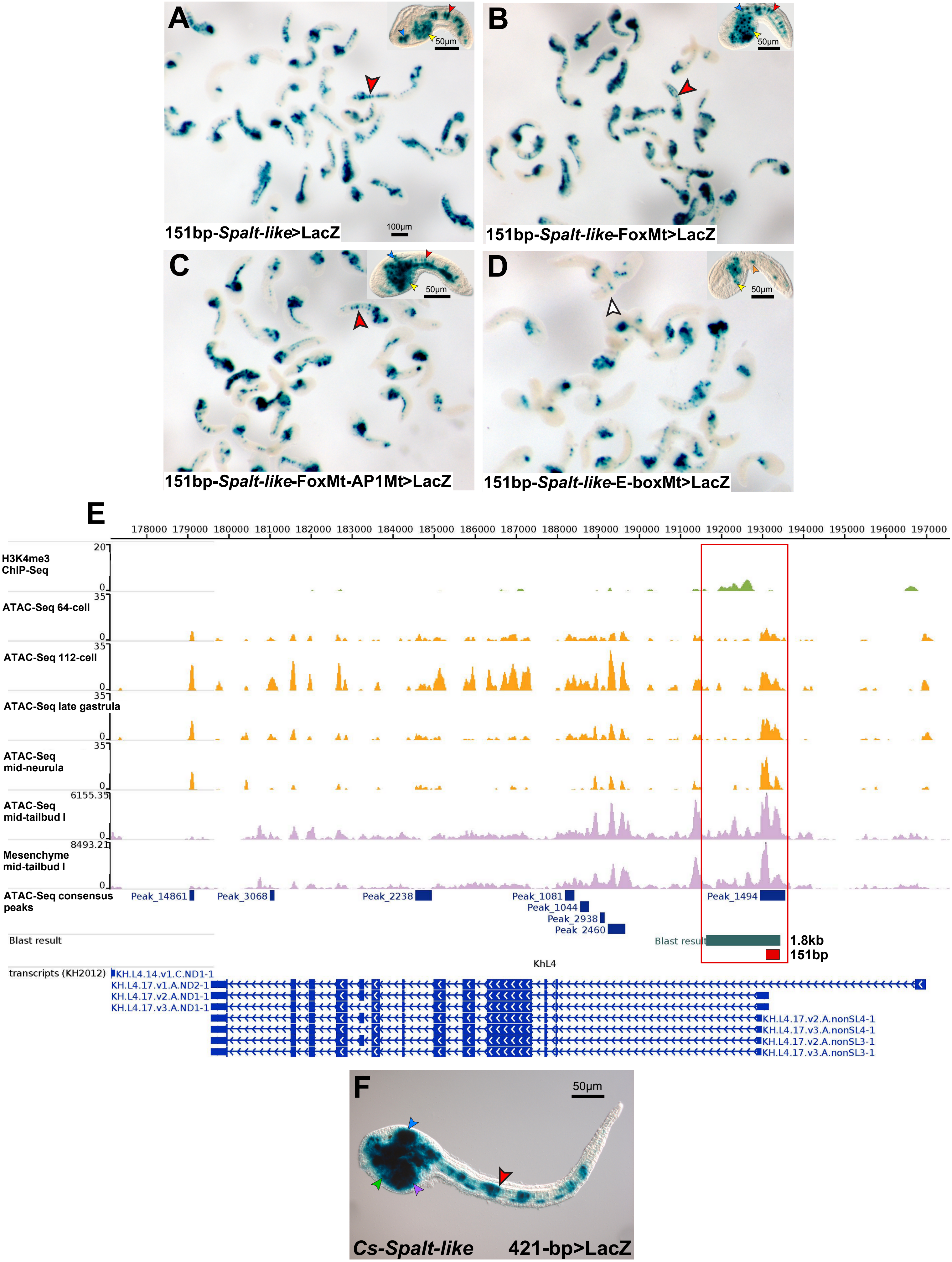
A
151bp-Spalt-like>LacZ
100μm
50μm
B
151bp-Spalt-like-FoxMt>LacZ
50μm
C
151bp-Spalt-like-FoxMt-AP1Mt>LacZ
50μm
D
151bp-Spalt-like-E-boxMt>LacZ
50μm
E
178000 179000 180000 181000 182000 183000 184000 185000 186000 187000 188000 189000 190000 191000 192000 193000 194000 195000 196000 197000
H3K4me3 ChIP-Seq
ATAC-Seq 64-cell
ATAC-Seq 112-cell
ATAC-Seq late gastrula
ATAC-Seq mid-neurula
ATAC-Seq mid-tailbud I
Mesenchyme mid-tailbud I
ATAC-Seq consensus peaks
Peak_14861
Peak_3068
Peak_2238
Peak_1081
Peak_1044
Peak_2938
Peak_2460
Peak_1494
Blast result
1.8kb
151bp
transcripts (KH2012)
KhL4
KH.L4.14.v1.C.ND1-1
KH.L4.17.v1.A.ND2-1
KH.L4.17.v2.A.ND1-1
KH.L4.17.v3.A.ND1-1
KH.L4.17.v2.A.nonSL4-1
KH.L4.17.v3.A.nonSL4-1
KH.L4.17.v2.A.nonSL3-1
KH.L4.17.v3.A.nonSL3-1
F
50μm
Cs-Spalt-like 421-bp>LacZ

Figure S11. Mutation analysis of the *Spalt-like* notochord CRM and overview of chromatin accessibility and interspecific conservation of the *Spalt-like* genomic locus.

(A-D) Low-magnification group microphotographs of *C. robusta* mid-tailbud embryos electroporated with the transgenes indicated in the lower left corner of each panel, fixed and X-gal stained. Insets: high-magnification microphotographs of representative embryos from each experiment. Arrowheads are color-coded as in Fig. 1. (E) Map of the active histone mark H3K4me3 ChIP-Seq plots (gastrula stage)[134] and chromatin accessibility landscape of the *Spalt-like* genomic locus, determined by ATAC-Seq experiments[52,53]. Green horizontal bar: the longest fragment with notochord activity identified in this study (1.8 kb); red rectangle: the 151-bp notochord CRM used for the mutation analysis. Blue gene models are from the Kyoto-Hoya (KH) gene assembly[135]; newest gene models (KY gene models[19]) are reported in Table 1. Screenshot in (E) reproduced with permission from Aniseed[132]. VISTA[64] comparisons did not identify sequence alignment in the region harboring the notochord CRM, likely due to gaps in the genome assemblies used by this computational tool; interspecific conservation of the binding site necessary for activity is reported in Table S3. (F) *C. robusta* late tailbud embryo carrying a *C. savignyi* genomic fragment corresponding to the *Spalt-like* notochord CRM (Fig. 5).

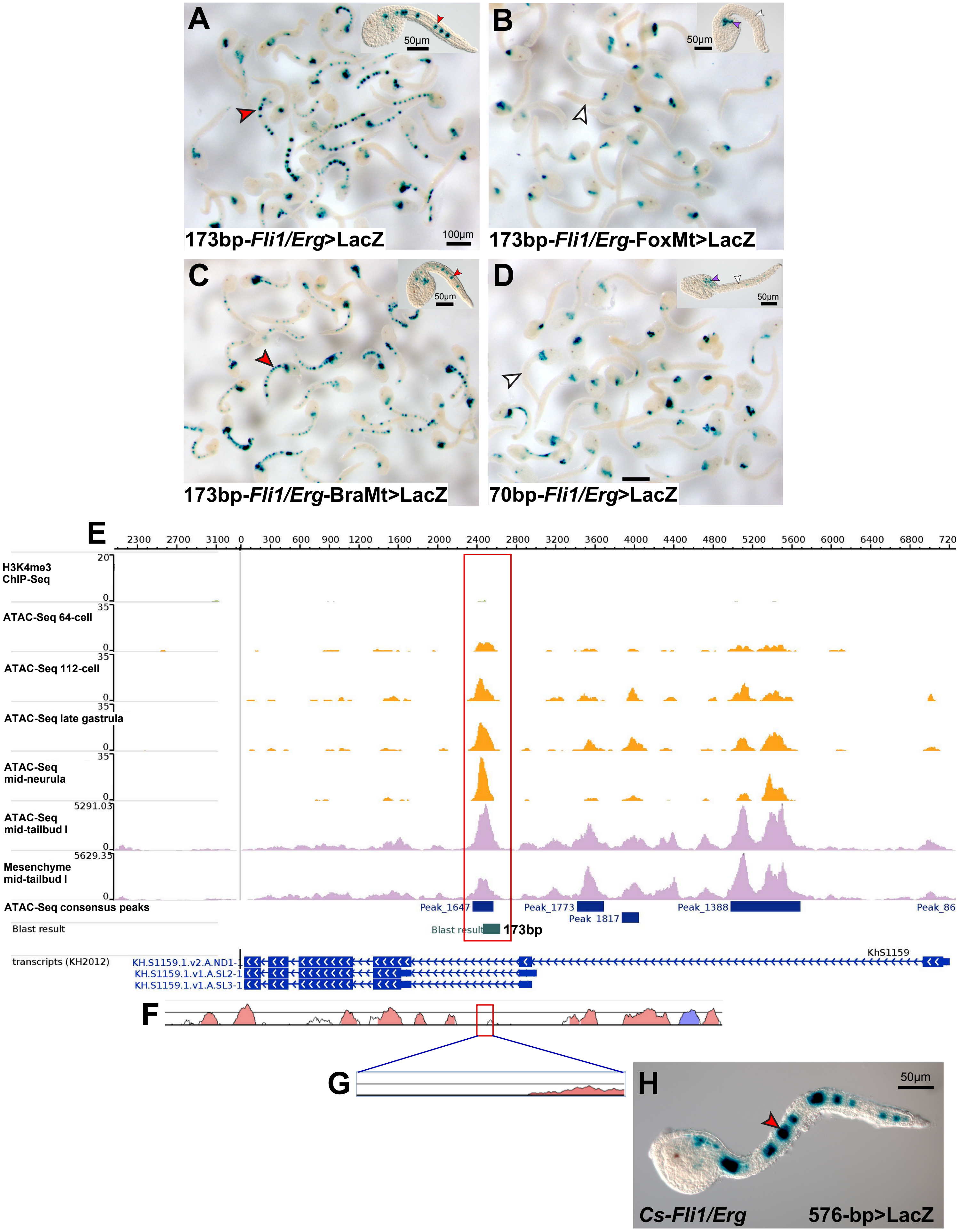

A
50µm
173bp-Fli1/Erg>LacZ
100µm
B
50µm
173bp-Fli1/Erg-FoxMt>LacZ
C
50µm
173bp-Fli1/Erg-BraMt>LacZ
D
50µm
70bp-Fli1/Erg>LacZ
E
H3K4me3 ChIP-Seq
ATAC-Seq 64-cell
ATAC-Seq 112-cell
ATAC-Seq late gastrula
ATAC-Seq mid-neurula
ATAC-Seq mid-tailbud I
Mesenchyme mid-tailbud I
ATAC-Seq consensus peaks
Peak_1647
Peak_1773
Peak_1817
Peak_1388
Peak_86
Blast result
173bp
transcripts (KH2012)
KH.S1159.1.v2.A.ND1-1
KH.S1159.1.v1.A.SL2-1
KH.S1159.1.v1.A.SL3-1
KhS1159
F
G
H
50µm
Cs-Fli1/Erg
576-bp>LacZ

Figure S12. Mutation analysis of the *Fli1/Erg* notochord CRM and overview of chromatin accessibility and interspecific conservation of the *Fli1/Erg* genomic locus.

(A-D) Low-magnification group microphotographs of *C. robusta* mid-tailbud embryos electroporated with the transgenes indicated in the lower left corner of each panel, fixed and stained for beta-galactosidase. Insets: high-magnification microphotographs of representative embryos from each experiment. Arrowheads are color-coded as in Fig. 1. (E) Map of the active histone mark H3K4me3 ChIP-Seq plots (gastrula stage)[134] and chromatin accessibility landscape of the *Fli1/Erg* genomic locus, determined by ATAC-Seq experiments[52,53]. Green horizontal bar: BLASTN result for the sequence of the 173-bp notochord CRM. Blue gene models are from the Kyoto-Hoya (KH) gene assembly[135]; newest gene models (KY gene models[19]) are reported in Table 1. Screenshot in (E) reproduced with permission from Aniseed[132]. (F) VISTA[64] plot of the sequence conservation between *C. robusta* and *C. savignyi*, reproduced with permission. Conserved non-coding regions are colored in pink, conserved coding regions in blue. (G) Close-up showing the conservation profile of the 173-bp *Fli1/Erg* notochord CRM. (H) *C. robusta* late tailbud embryo carrying a *C. savignyi* genomic fragment encompassing the region in (G).

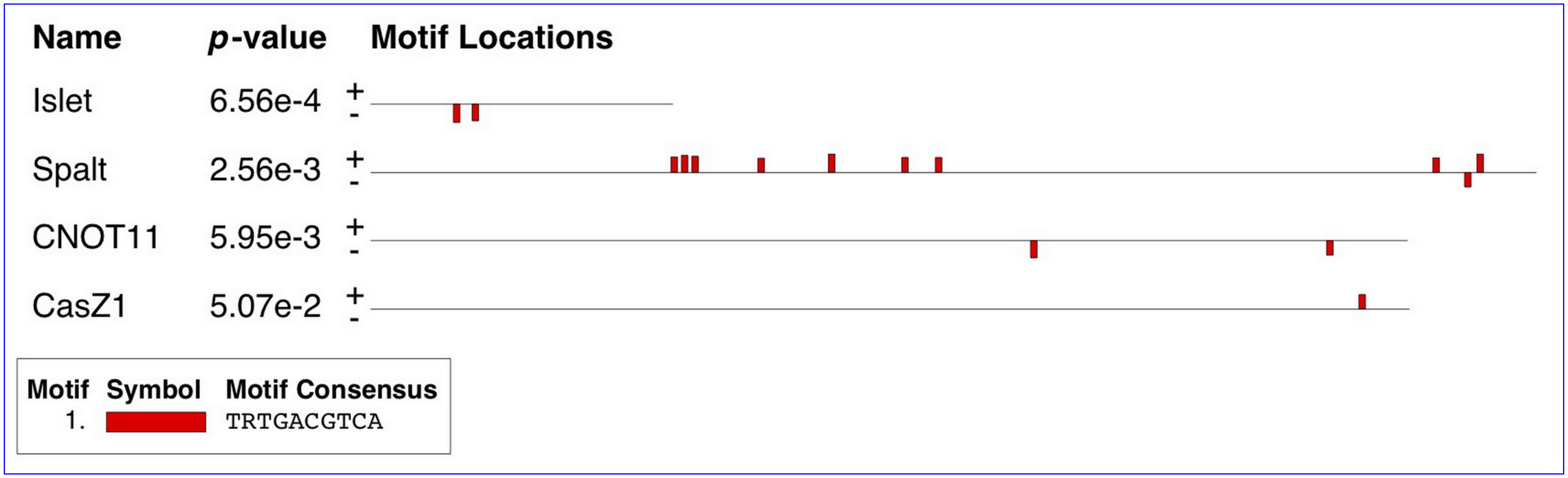

NAME	STRAND	START	P-VALUE	SITE
Islet1	-	129	7.16e-07	TGTGACGTCA
Islet1	-	158	2.59e-06	TGTGACGTGA
Spalt	+	1709	7.16e-07	TGTGACGTCA
Spalt	+	708	7.16e-07	TGTGACGTCA
Spalt	+	481	1.87e-06	TATGACGTCA
Spalt	+	497	3.75e-06	TATGACGTGA
Spalt	+	465	6.07e-06	GATGACGTCA
Spalt	+	873	9.10e-06	TATGACGTAA
Spalt	+	821	9.10e-06	TATGACGTAA
Spalt	+	1641	1.26e-05	TATGACGCGA
Spalt	-	1690	1.36e-05	TGTGTCGTCA
Spalt	+	599	1.75e-05	TGTGATGTCA
Cnot11	-	1020	1.87e-06	TATGACGTCA
Cnot11	-	1477	1.52e-05	GATGACGCCA
CasZ1	+	1527	1.63e-05	TATGACGTGC

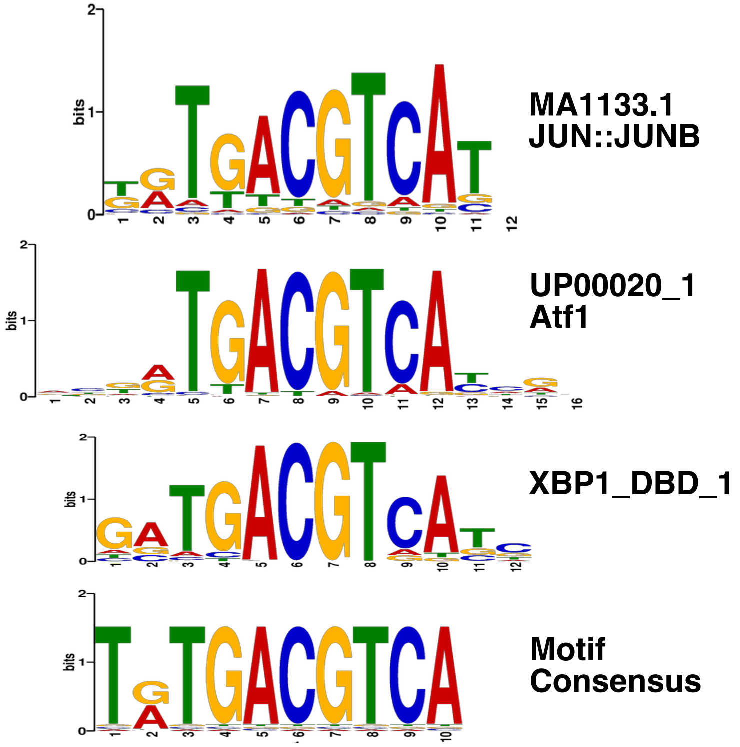

Figure S13. A statistically significant sequence motif related to the binding sites for TFs of the AP-1, CREB, and Xbp1 families, shared by four notochord CRMs.

(Top) Graphic output of MEME results, including p-value and location of the motif across four of the CRMs identified in this study (red rectangle). (Bottom, left panel) Coordinates, significance and individual sequences of each iteration of the motif. Sequences in green bold font are found within the 'minimal' 151-bp *Spalt-like* CRM. (Bottom, right panel) Comparison of the motif consensus with the consensus binding sites for JUN-JUNB dimers, Atf1, and XBP1, obtained through the TomTom software[137].

Table S1. CRMs genomic coordinates and distances from putative transcription start sites (TSS)

Gene name	Gene model used as a reference	CRM location	CRM coordinates	Distance from TSS*	TSS Cluster**	TSS cluster coordinates**
Aff4 (AF4/FRM2 family member 4)	KY21.Chr2.630.v1.SL1-1	Intron	Chr2:**3504559**..3504669	4.166 kb	TS_139315_SL	Chr2:3500364
CasZ1 (Castor Zinc Finger 1)	KY21.Chr1.1798.v1.ND1-1	Intron	Chr1:**12254851**..12254885	13.452 kb	TS_7352_nonSL	Chr1:12241377
Cnot11 (CCR4-Not transcription complex subunit 11)	KY21.Chr2.616.v1.SL1-1	Upstream	Chr2:3446653..**3446786**	-67 bp	TS_139127_SL	Chr2:3446882
Etv1 (Ets variant transcription factor 1)	KY21.Chr1.779.v1.SL2-1	Upstream	Chr1:**5573904**..5574053	-140 bp	TS_28529_SL	Chr1:5573755
Fli1/Erg (Ets-related gene)	KY21.Chr4.655.v1.SL1-1	Intron	Chr4:**4077693**..4077852	20.647 kb	TS_197264_SL	Chr4:4057218
Islet1 (ISL LIM homeobox)	KY21.Chr4.1164.v1.ND1-1	Upstream	Chr4:**7469569**..7469689	-82 bp	TS_207415_SL	Chr4:7469487
Lmx1-r (LIM homeobox transcription factor 1-related)	KY21.Chr9.611.v1.SL1-1	Upstream	Chr9:4378750..**4378877**	-844 bp	TS_294908_SL	Chr9:4379762
Mxd1 (MAX dimerization protein 1)	KY21.Chr1.459.v1.SL2-1	Intron	Chr1:**3333981**..3334192	1.098 kb	TS_22031_SL	Chr1:3332879
Ror-a (RAR-related orphan receptor)	KY21.Chr3.203.v2.SL2-1	Intron	Chr3:**1372844**..1373116	2.296 kb	TS_162060_SL	Chr3:1375524
Spalt-like	KY21.Chr5.1052.v1.nonSL1-1	Upstream	Chr5:**7099493**..7099630	-265 bp	TS_227834_nonSL	Chr5:7099215

*distance between the "minimal" CRM and the transcription start site (TSS), calculated using the coordinate in bold font.

**according to Brozovic et al., 2018, as reported on the Ghost website: http://ghost.zool.kyoto-u.ac.jp/default_ht.html

**Table S2. Functional Ci-Bra binding sites identified in this study.**		
**Sequence**	**Mechanism**	**CRM**
RWWNTNRCACYT*	Cooperative	*Drosophila orthopedia*
tTTCTCACACaa	Individual	*Aff4*
tTTTTAcCACCa	Individual	*Etv1*
cgTCTCACACac#	Individual	*Mxd1*
cTTTTTACACCa#	Individual	*Mxd1*
tAgCTGtCACgT	Cooperative	*ROR-a* site #5
tTgATGtCACgT	Cooperative	*ROR-a* site #6
AcTTTTcCACaT	Cooperative	*ROR-a* site #7
cccGTTtCACaa	Individual	*Cnot11*
cATTTCACACgc	Individual**	*Islet1*

Bases that diverge from the consensus are indicated in lower case. The core TNNCAC sequence is underlined.
N: any base; R: A or G; W: A or T; Y: C or T.

* Kusch T, Storck T, Walldorf U, Reuter R. Brachyury proteins regulate target genes through modular binding sites in a cooperative fashion. *Genes Dev*. 2002. 16(4):518-29. See also Katikala et al., 2013 for additional *Ciona* binding sites.

**This binding site is required along with a homeodomain binding site.

#These binding sites are arranged as a quasi-palindrome and seem to function as an individual site, since mutations in either one can obliterate notochord expression.

Table S3. Interspecific conservation of the activator binding sites identified in this study.

CRM	Main activator consensus TFBS	*C. robusta* main activator TFBS	*C. savignyi* sequence	*C. savignyi* match to consensus TFBS
Lmx1-r	Fox TRTTTRY	TGTTTGT	TGTgTGT	No
Aff4	T-box TNNCAC	TCACAC	TGGtAC	No
Etv1	T-box TNNCAC	TACCAC	TGCCAC	Yes
Mxd1	T-box TNNCAC	TCACAC	TCCCAC	Yes
Mxd1	T-box TNNCAC	TTACAC	TTACAC	Yes
Ror-a	T-box TNNCAC	TGTCAC	TGTtAC	No
Ror-a	T-box TNNCAC	TGTCAC	cGTaAa	No
Ror-a	T-box TNNCAC	TTCCAC	TCCgCC	No
CasZ1	Fox TRTTTRY	TGTTTAA	TGTgTTC	No
CasZ1	AP-1 TGAC	TGAC	TGAC	Yes
Cnot11	Fox TRTTTRY	TGTTTAC	cGcTTcT	No
Cnot11	T-box TNNCAC	TTTCAC	no alignment	No
Islet1	Homeodomain TAAT	TAAT	TAAT	Yes
Islet1	T-box TNNCAC	TCACAC	TCACAC	Yes
Spalt-like	E-box CANNTG	CAATTG	tgATTG	No
Fli/Erg	Fox TRTTTRY	TGTTTGC	TGTTTGT	Yes

Table S4. Oligonucleotide primers utilized for the identification and characterization of the notochord CRMs analyzed in this study.		
**Construct name, size, and ATAC-Seq peak(s) associated with it**	**Forward Primer** (5'→3') Restriction site: XhoI (ctcgag)	**Reverse Primer** (5'→3') Restriction site: XbaI (tctaga)
***Aff4* Notochord CRM**		
**1. Identification of the notochord enhancer region**		
UR#1 (1923bp) ATAC-Seq Peak: 12873	1923-F acgtctcgagTTGCCAATGTAACAGTTTTGTG	1923-R acgttctagaTCTGGTGAATTGGGTTTTGTC
Intron 2 #1 (558bp) ATAC-Seq Peak: 1389	558-F acgtctcgagTATGTTTACGCAGTAGCGCC	558-R acgttctagaATAGAACTTTAGTGCGGATGTTGA
Intron 2 #2 (1117bp) ATAC-Seq Peaks: 1978, 1610	1117-F acgtctcgagCTGCAGTAACAGCAATCTCGG	1117-R acgttctagaTCAATATGACGCAACGACGC
Intron 2 #3 (1245bp) ATAC-Seq Peaks: 3236, 3306, 1220	1245-F acgtctcgagTCACTTTGACGAATCGCTGC	1245-R acgttctagaAACTGGTGGCATTGTGACGTA
Intron 2 #4 (1170bp) ATAC-Seq Peak: 1211	1170-F acgtctcgagGAGTCAGTTGTGCAAGCGAT	1170-R acgttctagaGGTCGGCTAAAGCAAGCATA
UR#2 (1844bp)	1844-F acgtctcgagGAAGGACCGCATAGCCGTGT	1844-R acgttctagaGGCATCTTATACGGTTGCCTGAATTG
**2. Truncations of the 1117-bp notochord CRM**		
Intron 2 #2, 1-523 (520bp) ATAC-Seq Peak: 1978	1117-F acgtctcgagCTGCAGTAACAGCAATCTCGG	520-R acgttctagaATAGAAACGGGTGCGTCAAC
Intron 2 #2, 495-1117 (625bp) ATAC-Seq Peak: 1610	625-F acgtctcgagTTCGATACGTTGACGCACCC	1117-R acgttctagaTCAATATGACGCAACGACGC
Intron 2 #2, 806-1117 (315bp) ATAC-Seq Peak: 1610	315-F acgtctcgagATACGGCTTGCAGAGAGCAT	1117-R acgttctagaTCAATATGACGCAACGACGC
Intron 2 #2, 806-941 (136bp) ATAC-Seq Peak 1610	315-F acgtctcgagATACGGCTTGCAGAGAGCAT	136-R acgttctagaTCTCACACAATATAAAATTGCGACG
Intron 2 #2, 905-1117 (216bp)	216-F	1117-R

ATAC-Seq Peak: 1610	acgtctcgagTGTTTAAACGTT CGTCGCAAT	acgttctagaTCAATATGACGCAA CGACGC
Intron 2 #2, 905-1069 (168bp)<br>ATAC-Seq Peak: 1610	216-F<br>acgtctcgagTGTTTAAACGTT CGTCGCAAT	168-R<br>atgctctagaTCAATAAAGTTAAC CTCAGCGACG
Intron 2 #2, 897-1041 (149bp)<br>ATAC-Seq Peak: 1610	149-F<br>acgtctcgagCAAATTGTTTAA ACGTTCGTCGCA	140-R<br>acgttctagaGATTTGTTTACACGT AAAGTTGGC
Intron 2 #2, 917-1044 (128bp)<br>ATAC-Seq Peak: 1610	128-F<br>acgtctcgagCGTCGCAATTTT ATATTGTGTGAG	140-R<br>acgttctagaGATTTGTTTACACGT AAAGTTGGC
Intron 2 #2, 905-1006 (102bp)<br>ATAC-Seq Peak: 1610	216-F<br>acgtctcgagTGTTTAAACGTT CGTCGCAAT	102-R<br>acgttctagaGATGGTGCAAGTCA CACGG
**3. Mutation Analysis**		
Intron 2 #2, 905-1069 (168bp)<br>CREB Mt	216-F<br>acgtctcgagTGTTTAAACGTT CGTCGCAAT	168-CREB-Mt-R<br>atgctctagaTCAATAAAGTTAAC CTCAGCtgatGCATGATTTG
Intron 2 #2, 897-1041 (149bp)<br>Bra 1 MT	149-Bra1-Mt-F<br>acgtctcgagCAAATTGTTTAA ACGTTCGTCGCAATTTTAT ATTagaTGAGAAAACTCAC	140-R<br>acgttctagaGATTTGTTTACACGT AAAGTTGGC
Intron 2 #2, 897-1041 (149bp)<br>Fox Mt	149-Fox-Mt-F<br>acgtctcgagCAAATTGgggAAA CGTTCGTCGCA	140-R<br>acgttctagaGATTTGTTTACACGT AAAGTTGGC
Intron 2 #2, 897-1041 (149bp)<br>Bra and Fox Mt	149-Bra1+Fox-Mts-F<br>acgtctcgagCAAATTGgggAAA CGTTCGTCGCAATTTTATA TTagaTGAGAAAACTCAC	140-R<br>acgttctagaGATTTGTTTACACGT AAAGTTGGC
Intron 2 #2, 905-1006 (102bp)<br>Bra 2 and Bra 3 Mt	216-F<br>acgtctcgagTGTTTAAACGTT CGTCGCAAT	102-Bra2+Bra3-Mt-R<br>acgttctagaGATGagaCAAGTCAtct G
CasZ1 **Notochord CRM**		
**1. Identification of the notochord enhancer region**		
UR #1 (1,285bp)	1285-F<br>acgtctcgagACAATCTGTGTT GCATTTCCC	1285-R<br>acgttctagaTGTTTCTAGCGCCTT TACCG
UR #2 (1,190bp)<br>ATAC-Seq Peak: 771	1190-F<br>acgtctcgagATTTATTCTTCAC CAACACGCCG	1190-R<br>acgttctagaTCAAAACTAGCTCC CTGCCAA
UR #3 (1,604bp)<br>ATAC-Seq Peaks: 774, 769	1604-F<br>acgtctcgagGCCCATCAACCT TATCTCGC	1604-R<br>acgttctagaCACCGCAAACCGTG TTTTACT
Int 1 (1,005bp)	10005-F	1005-R

	acgtctcgagGAGTCCGAACACCCTGGAAA	acgttctagaCATTGGGGTGATAAGCCGGT
**2. Truncations of the 1604-bp Notochord CRM**		
UR #3, 1-430 (430bp)<br>ATAC-Seq Peaks: 774, 769	1604-F<br>acgtctcgagGCCCATCAACCTTATCTCGC	430-R<br>acgttctagaTCTCATTTAGAGCGCTGACC
UR #3, 394-1048 (665bp)<br>ATAC-Seq Peaks: 769	665-F<br>acgtctcgagGCCGCAAGGTTTAATTGGGT	665-R<br>acgttctagaGCACATTAGCGCTATACAACTGC
UR #3, 394-1048 (665bp)<br>ATAC-Seq Peaks: 769	665-F<br>acgtctcgagGCCGCAAGGTTTAATTGGGT	665-R<br>acgttctagaGCACATTAGCGCTATACAACTGC
UR #3, 394-723 (330bp)<br>ATAC-Seq Peaks: 769	665-F<br>acgtctcgagGCCGCAAGGTTTAATTGGGT	330-R<br>acgttctagaAATCCAGAACCTCAAAAGCCG
UR #3, 699-1048 (350bp)	350-F<br>acgtctcgagTAGACGGCTTTTGAGGTTCTGG	665-R<br>acgttctagaGCACATTAGCGCTATACAACTGC
UR #3, 699-868 (170bp)	350-F<br>acgtctcgagTAGACGGCTTTTGAGGTTCTGG	170-R<br>acgttctagaGCACCGAGTGCCGAAAT
UR #3, 848-1048 (201bp)	201-F<br>acgtctcgagCGACATTTCGGCACTCGGT	665-R<br>acgttctagaGCACATTAGCGCTATACAACTGC
UR #3, 770-933 (164bp)	164-F<br>acgtctcgagTCGACGCTGAAAGGAAGTCA	164-R<br>acgttctagaATTGAGCTGCGCCAATAACG
UR #3, 794-933 (140bp)	140-F<br>acgtctcgagTTCAGCCTTCTGTTCCAAAATGT	164-R<br>acgttctagaATTGAGCTGCGCCAATAACG
UR #3, 806-933 (128bp)	128-F<br>acgtctcgagTTCCAAAATGTGTGCTTGACTG	164-R<br>acgttctagaATTGAGCTGCGCCAATAACG
UR #3, 813-933 (121bp)	121-F<br>acgtctcgag ATGTGTGCTTGACTGTTTAACTTGA	164-R<br>acgttctagaATTGAGCTGCGCCAATAACG
UR #3, 838-933 (96bp)	96-F<br>acgtctcgagCGTGTTCCCGCGACAT	164-R<br>acgttctagaATTGAGCTGCGCCAATAACG
**3. Mutation Analysis**		
UR #3, 806-933 (128bp)<br>Zinc finger mutant	128-Zinc-MT-F<br>acgtctcgagTTCCAAAcctctcGCTTGACTG	164-R<br>acgttctagaATTGAGCTGCGCCAATAACG

UR #3, 813-933 (121bp)<br>Fox mutant	121-Fox-Mt-F<br>acgtctcgagATGTGTGCTTGACTGgggAACTTGAcgtgttcc	164-R<br>acgttctagaATTGAGCTGCGCCAATAACG
UR #3, 813-933 (121bp)<br>AP1 mutant	121-AP1-Mt-F<br>acgtctcgagATGTGTGCTTGACTGTTTAACTgtcCGTGTTCCCGCGACA	164-R<br>acgttctagaATTGAGCTGCGCCAATAACG
***Cnot11* Notochord CRM**		
**1. Identification of the notochord enhancer region**		
UR #1, -1569 to -66 (1504bp)<br>ATAC-Seq Peak: 8072	1504-F<br>acgtctcgagGCTCAAATGAGCAGTTTTTCAG	1504-R<br>acgttctagaTCCTCCACTACCACTTCGATTTG
**2. Truncations**		
UR #1, 1-770 (770bp)	1504-F<br>acgtctcgagGCTCAAATGAGCAGTTTTTCAG	770-R<br>acgttctagaCAGCACAGCTGAAGTTATGGAC
UR #1, 755-1504 (750bp)<br>ATAC-Seq Peak: 8072	750-F<br>acgtctcgagAACTTCAGCTGTGCTGCTTTA	1504-R<br>acgttctagaTCCTCCACTACCACTTCGATTTG
UR #1, 755-1124 (370bp)	750-F<br>acgtctcgagAACTTCAGCTGTGCTGCTTTA	370-R<br>acgttctagaTAAACCTCACTGGTTTTCGCA
UR #1, 1104-1504 (401bp)<br>ATAC-Seq Peak: 8072	401-F<br>acgtctcgagTGCGAAAACCAGTGAGGTTTA	1504-R<br>acgttctagaTCCTCCACTACCACTTCGATTTG
UR #1, 1347-1504 (158bp)<br>ATAC-Seq Peak: 8072	158-F<br>acgtctcgagTCTATGTAGCGCTGGTGAAGC	1504-R<br>acgttctagaTCCTCCACTACCACTTCGATTTG
UR #1, 1347-1476 (130bp)<br>ATAC-Seq Peak: 8072	158-F<br>acgtctcgagTCTATGTAGCGCTGGTGAAGC	130-R<br>acgttctagaATGACGCCAGTAACACACTTT
UR #1, 1347-1462 (116bp)	158-F<br>acgtctcgagTCTATGTAGCGCTGGTGAAGC	116-R<br>acgttctagaACACTTTCCAACTGACCCCG
UR #1, 1347-1445 (101bp)	158-F<br>acgtctcgagTCTATGTAGCGCTGGTGAAGC	101-R<br>acgttctaGACCCCGTTTCACAGGTGAACTA
**3. Mutation Analysis**		
1347-1504 (158bp)<br>Bra 3 Mt	158-F<br>acgtctcgagTCTATGTAGCGCTGGTGAAGC	1504-Bra3-Mt-R<br>acgttctagaTCCTCCACTACtctTTCGATTTG
1347-1504 (158bp)<br>Bra 1 Mt	158-F<br>acgtctcgagTCTATGTAGCGCTGGTGAAGC	106-Bra1-Mt-R<br>ACTGACCCCGTggtACAAGTGAACTAGT

Overlapping PCR	80-Bra1-Mt-F<br>ACTAGTTCACTTGTaccACG GGGTCAGT	1504-R<br>acgttctagaTCCTCCACTACCACT TCGATTTG
1347-1504 (158bp)<br>Bra 2 Mt<br>Overlapping PCR	158-F<br>acgtctcgagTCTATGTAGCGC TGGTGAAGC<br>58-Bra2-Mt-F<br>GTCAGTTGGAAAGTagaTT ACTGGCGTCATCA	132-Bra2-Mt-R<br>TGATGACGCCAGTAAtctACTT TCCAACTGAC<br>1504-R<br>acgttctagaTCCTCCACTACCACT TCGATTTG
1347-1504 (158bp)<br>Fox Mt<br>Overlapping PCR	158-F<br>acgtctcgagTCTATGTAGCGC TGGTGAAGC<br>100-Fox-Mt-F<br>TTTTTTGTTCTGcccACAAA TCGCAGT	70-Fox-Mt-R<br>CCAACCTAACTGCGATTTGTg ggCAGAACAAAAAAGGC<br>1504-R<br>acgttctagaTCCTCCACTACCACT TCGATTTG
1347-1504 (158bp)<br>AP1 Mt<br>Overlapping PCR	158-F<br>acgtctcgagTCTATGTAGCGC TGGTGAAGC<br>58-AP1-Mt-F<br>GTCAGTTGGAAAGTGTGT TACTGGCtttATCATAACAA ATCG	142-AP1-Mt-R<br>CGATTTGTTATGATaaaGCCAG TAACACACT<br>1504-R<br>acgttctagaTCCTCCACTACCACT TCGATTTG
Etv1 **Notochord CRM**		
**1. Identification of the notochord enhancer region**		
Intron 1 (665bp)	665-F<br>acgtctcgagTCTGGTTATTGG ACGGCTGTT	665-R<br>acgttctagaTGCGTACACTTTGTC ATAATGAAT
UR (1650bp)	1650-F<br>acgtgtcgacCCTAAACACGAT GCACACAG	1650-R<br>acgtccatggTATTCTCACGGCCC AACAG
**2. Truncations**		
Intron 1, 1-351 (351bp)	665-F<br>acgtctcgagTCTGGTTATTGG ACGGCTGTT	351-R<br>acgttctagaTTCTGCCTCGTACAC ACACG
Intron 1, 332-655 (324bp)	324-F<br>acgtctcgagCGTGTGTGTACG AGGCAGAA	660-R<br>acgttctagaTGCGTACACTTTGTC ATAATGAAT
Intron 1, 332-503 (171bp)	324-F<br>acgtctcgagCGTGTGTGTACG AGGCAGAA	171-R<br>acgttctagaATAGAGAAGCTGTG TTTGCAGTTT
Intron 1, 478-655 (178bp)	178-F<br>acgtctcgagAAAACTGCAAAC ACAGCTTCTCT	665-R<br>acgttctagaTGCGTACACTTTGTC ATAATGAAT

Intron 1, 497-655 (159bp)	159-F<br>acgtctcgagCTCTATTTTTACCACCAAAGTGTTTTGTG	665-R<br>acgttctagaTGCGTACACTTTGTCATAATGAAT
**3. Mutation Analysis**		
497-655 (159bp)<br>Bra1 Mt	159-Bra1-Mt-F<br>acgtctcgagCTCTATTTTTACtctCAAAGTGTTTTGTG	665-R<br>acgttctagaTGCGTACACTTTGTCATAATGAAT
***Fli/Erg* Notochord CRM**		
**1. Identification of the notochord enhancer region**		
UR #1,-58 to 1292 (1358bp)<br>ATAC-Seq Peak: 8641	1358-F<br>acgtctcgagCAAAGTTCTTTGCGTGGACAC	1358-R<br>acgtctgcagAATGTCGGTCCAATTCCAAC
UR #2, 51 to 985 (934bp)<br>ATAC-Seq Peaks: 1773, 1817	934-F<br>acgtgtcgacGTTTAACTGGCCCACTACAAAGT	934-R<br>acgtccatggCCTTGCCGTTCTGGTGATAC
Int 1 (1370bp)<br>ATAC-Seq Peak: 1645	1370-F<br>acgtctcgagATTGGGCGCAGTGTATCTGT	1370-R<br>acgttctagaAGCTTAGTACTTTGTAGTGGGC
**2. Truncations**		
Int 1, 739-1370 (632bp)<br>ATAC-Seq Peak: 1645	632-F<br>acgtctcgagCGTACCGCTAACGTTACACCA	1370-R<br>acgttctagaAGCTTAGTACTTTGTAGTGGGC
Int 1, 867-1370 (504bp)<br>ATAC-Seq Peak: 1645	504-F<br>acgtctcgagTGCGAATTTACACCCACTTGCT	1370-R<br>acgttctagaAGCTTAGTACTTTGTAGTGGGC
Int 1, 867-1279 (413bp)<br>ATAC-Seq Peak: 1645	504-F<br>acgtctcgagTGCGAATTTACACCCACTTGCT	413-R<br>acgttctagaTCCAAGCTAAGCCTAAATTTGTTT
Int 1, 867-1182 (316bp)<br>ATAC-Seq Peak: 1645	504-F<br>acgtctcgagTGCGAATTTACACCCACTTGCT	316-R<br>acgttctagaACCACCAGCGACGTTTAAAGA
Int 1, 1127-1279 (153bp)<br>ATAC-Seq Peak: 1645	153-F<br>acgtctcgagATTGTTTTGTGTTCTTTGCTTGC	413-R<br>acgttctagaTCCAAGCTAAGCCTAAATTTGTTT
Int 1, 604-883 (280bp)<br>ATAC-Seq Peak: 1645	280-F<br>acgtctcgagTTTGCAGACGCAAACTCGCT	280-R<br>acgttctagaGTGGGTGTAAATTCGCAAGAGT
Int 1, 747-919 (173bp)<br>ATAC-Seq Peak: 1645	173-F<br>acgtctcgagTAACGTTACACCATGTTTGCATAGCCC	173-R<br>acgttctagaATAAATGAATGCACCATGTTTATGT
Int 1, 739-884 (145bp)<br>ATAC-Seq Peak: 1645	632-F<br>acgtctcgagCGTACCGCTAACGTTACACCA	280-R<br>acgttctagaGTGGGTGTAAATTCGCAAGAGT

Int 1, 850-919 (70bp)<br>ATAC-Seq Peak: 1645	70-F<br>acgtctcgagACAGTACAAGAA ACTCTTGCGA	173-R<br>acgttctagaATAAATGAATGCAC CATGTTTATGT
**3. Mutation Analysis**		
747-919 (173bp)<br>Bra1 Mt	173-Bra-Mt-F<br>acgtctcgagTAACGTTcttaCAT GTTTGCATAGCCC	173-R<br>acgttctagaATAAATGAATGCAC CATGTTTATGT
747-919 (173bp)<br>Fox Mt	173-Fox-Mt-F<br>acgtctcgagTAACGTTACACC AaGagtGCATAGCCC	173-R<br>acgttctagaATAAATGAATGCAC CATGTTTATGT
***Islet1* Notochord CRM**		
**1. Identification of the notochord enhancer region**		
UR #1, -304 to -165 (140bp)	140-F<br>acgtctcgagTATACGCGTGTG GGGAGGTT	140-R<br>atgctctagaCCAAATATTCCTCG ATGTGGTGA
**2. Truncations**		
UR #1, 8-140 (133bp)	133-F<br>acgtctcgagGTGTGGGGAGGT TAATTAAC	140-R<br>atgctctagaCCAAATATTCCTCG ATGTGGTGA
UR #1, 16-140 (125bp)	125-F<br>acgtctcgagAGGTTAATTAAC ATGGGCG	140-R<br>atgctctagaCCAAATATTCCTCG ATGTGGTGA
UR #1, 28-102 (75bp)	75-F<br>acgtctcgagATGGGCGTGTGA AATGTAATTAG	75-R<br>acgttctgagGCCTACAGTGCAAA TGAATGCCTTC
**3. Mutation Analysis**		
16-140 (125bp)<br>HD Mt	125-HD-Mt-F<br>acgtctcgagAGGTTccggAACA TGGGCG	140-R<br>atgctctagaCCAAATATTCCTCG ATGTGGTGA
16-140 (125bp)<br>Bra 1 Mt	125-Bra1-Mt-F<br>acgtctcgagAGGTTAATTAAC ATGGGCagaTGAAATGTAA TTAGTTAA	140-R<br>atgctctagaCCAAATATTCCTCG ATGTGGTGA
16-140 (125bp)<br>Bra 2 Mt<br>Overlapping PCR	60-Bra2-Mt-F<br>GGCATTCATTTGtctTGTAG GCATTGAAC<br>125-F<br>acgtctcgagAGGTTAATTAAC ATGGGCG	140-R<br>atgctctagaCCAAATATTCCTCG ATGTGGTGA<br>95-Bra2-Mt-R<br>AATGCCTACAagaCAAATGAA TG
***Lmx1-r* Notochord CRM**		
**1. Identification of the notochord enhancer region**		
UR #1 (1203bp)	1203-F	1203-R

	acgtctcgagTTTTGCCGGCTGGTAAGACC	acgttctagaTCTGGCTTTTTCAAGATAATTCG
**2. Truncations**		
UR #1 1-592 (592bp)	1203-F<br>acgtctcgagTTTTGCCGGCTGGTAAGACC	592-R<br>acgttctagaCTATGATTCCCCTAGGCCGT
UR #1, 564-1203 (640bp)	640-F<br>acgtctcgagCAATTAAAAACGGCCTAGGGG	1203-R<br>acgttctagaTCTGGCTTTTTCAAGATAATTCG
UR #1, 1234-525 (292bp)	292-F<br>acgtctcgagTGCCGTTCTACTTGTAGCTGA	292-R<br>acgttctagaAATGTGTCCTGCCTCCCCC
UR #1, 234-402 (169bp)	292-F<br>acgtctcgagTGCCGTTCTACTTGTAGCTGA	169-R<br>acgttctagaACGTTAGCGATGACCTCAACTT
UR #1, 258-402 (145bp)	145-F<br>acgtctcgagACGTATTTACGTTGGAGATTTG	169-R<br>acgttctagaACGTTAGCGATGACCTCAACTT
UR #1, 278-402 (125bp)	125-F<br>acgtctcgagTGACGTCAACCATTCGACTT	169-R<br>acgttctagaACGTTAGCGATGACCTCAACTT
UR #1, 294-402 (109bp)	109-F<br>acgtctcgagACTTGTGTGCTAGCGGCAG	169-R<br>acgttctagaACGTTAGCGATGACCTCAACTT
UR #1 306-402 (97bp)	97-F<br>acgttctagaGCGGCAGTTCAACAACG	169-R<br>acgttctagaACGTTAGCGATGACCTCAACTT
UR #1 234-322 (89bp)	292-F<br>acgtctcgagTGCCGTTCTACTTGTAGCTGA	89-R<br>acgtctcgagCGTTGTTGAACTGCCGC
**3. Mutation Analysis**		
UR #1, 234-402 (169bp)<br>Fox Mt	63-Fox-Mt-F<br>CAAATGCACGCAACTTtgggGTGTTG<br>292-F<br>acgtctcgagTGCCGTTCTACTTGTAGCTGA	169-R<br>acgttctagaACGTTAGCGATGACCTCAACTT<br>142-Fox-Mt-R<br>CTTCATTGAGCAACACcccaAAGTT
UR #1, 234-402 (169bp)<br>Bra Mt	292-F<br>acgtctcgagTGCCGTTCTACTTGTAGCTGA<br>114-Bra-Mt-F<br>ATTCGACTTGTtctCTAGCGGCAG	79-Bra-Mt-R<br>CTGCCGCTAGagaACAAGTCGAAT<br>169-R<br>acgttctagaACGTTAGCGATGACCTCAACTT

UR #1, 306-402 (97bp)<br>Fox Mt<br>Overlapping PCR	97-F<br>acgttctagaGCGGCAGTTCAACAACG<br>63-Fox-Mt-F<br>CAAATGCACGCAACTTtgggGTGTTG	142-Fox-Mt-R<br>CTTCATTGAGCAACACcccaAAGTT<br>169-R<br>acgttctagaACGTTAGCGATGACCTCAACTT
Mxd1/Noto7 **Notochord CRM**		
**1. Identification of the notochord enhancer region**		
UR #1, -951 to +43 (995bp)<br>ATAC-Seq Peak 9394	995-F<br>atcgctcgagTGTACTCTTGAGGCACTTTTAGGA	995-R<br>atgctctagaAGGCAGCGTAAAACTACAAACACA
Intron 2 #1 (1000bp)<br>ATAC-Seq Peaks: 2248, 2516	1000-F<br>atcgctcgagGGGAAGACCATGAATGTTTACCG	1000-R<br>atgctctagaTCTCTTTTGTGCGTACAACAACTC
Intron 2 #2 (1203bp)<br>ATAC-Seq Peak: 1737	1203-F<br>atcgctcgagAGTTTTCTACGTCATGTCGAAGTTT	1203-R-wt<br>atgctctagaGGGTTTCCAGGGCACATGAT
Intron 2 #3 (1101bp)	1101-F<br>atcgctcgagCACGCTGCCACACGTAATG	1101-R<br>atgctctagaTTGTGATACATAATAAAAGCGGGAG
Intron 3 #1 (853bp)<br>ATAC-Seq Peaks: 2513, 2798	853-F<br>atcgctcgagACGTGTATTGTTTGGCTGTGTT	853-R<br>atgctctagaCAGTTAAGTGCCGGGATAACG
Intron 3 #2 (1025bp)<br>ATAC-Seq Peak: 2004	1025-F<br>atcgctcgagCGTGGCCAACATTGGCTAAAT	1025-F<br>atgctctagaGCATGTAAACACCAGTGTGGATT
DR #1, 10391 to 11374 (534bp)<br>ATAC-Seq Peak: 14356	10391-F<br>atcgctcgagACCTTAAGTTTCGTTGCCGGGT	11374-R<br>atgctctagaTCCCGAAGCTGTGGCAAAGTGC
**2. Truncations**		
Intron 2 #1, 560-1000 (440bp)<br>ATAC-Seq Peak: 2516	440-F<br>atcgctcgagTCGTCTCCCAAATACATCGCA	1000-R<br>atgctctagaTCTCTTTTGTGCGTACAACAACTC
Intron 2 #1, 91-307 (216bp)<br>ATAC-Seq Peak: 2248	216-F<br>acgtctcgagCGTCTCACACACTGGTGTAAAAAGGCGTGTC	216-R<br>acgttctagaGCTTTAACACCCCCTGCAGATACGCAAG
**3. Mutation Analysis**		
91-307 (216bp)<br>Bra1 Mt	216-Bra1-Mt-F<br>acgtctcgagCGTCTCAtctACTGGTGTAAAAAGGCGTGTC	216-R<br>acgttctagaGCTTTAACACCCCCTGCAGATACGCAAG
91-307 (216bp)	216-Bra2-Mt-F	216-R

Bra2 Mt	acgtctcgagCGTCTCACACACTGtaaTAAAAAGGCGTGTC	acgttctagaGCTTTAACACCCCCTGCAGATACGCAAG
91-307 (216bp)<br><br>Bra3 Mt	216-F<br>acgtctcgagCGTCTCACACACTGGTGTAAAAAGGCGTGTC	216-Bra3-Mt-R<br><br>acgttctagaGCTTTAAtgtCCCCTGCAGATACGCAAG
***Ror-a* Notochord CRM**		
**1. Identification of the notochord enhancer region**		
UR #1 (1800bp)<br>ATAC-Seq peaks: 987, 1792	1800-F<br>acgtctcgagCCTCTCTGGATACTCCACGTT	1800-R<br>acgttctagaAGGCTACCTATCTTACCCAAAG
UR #2 (500bp)	500-F<br>acgtctcgagACGATACGCATCTCGCCAA	500-R<br>acgttctagaAGCGAAGATAAATCATAAACGCAA
Int 1 (1122bp)<br>ATAC-Seq Peak: 1560	1122-F<br>acgtctcgagTGAGCTGAAATGACAAGGTTCAG	1122-R<br>acgttctagaATCCCAGCCCGTGGTAAGTT
Int 3 (530bp)<br>ATAC-Seq Peak: 1385	530-F<br>acgtctcgagTTAAGCTAAATTGGCGACCGA	530-R<br>acgttctagaAGCGTTTGTTCAGTTCTCAGTG
Int 2 (953bp)<br>ATAC-Seq Peaks: 2801, 2461, 2541	953-F<br>acgtctcgagGAATGCGTGTGAATCGCTCA	953-R<br>acgttctagaTTCCAGCCACCCCTTAAAGT
**2. Truncations**		
Int 2, 1-520 (520bp)<br>ATAC-Seq Peaks: 2801, 2461	953-F<br>acgtctcgagGAATGCGTGTGAATCGCTCA	520-R<br>acgttctagaGCCATTGTGTGGTATCCATCC
Int 2, 392-953 (562bp)<br>ATAC-Seq Peaks: 2461, 2541	562-F<br>acgtctcgagCGCAATGCAGCAAGCGATG	953-R<br>acgttctagaTTCCAGCCACCCCTTAAAGT
**3. Mutation Analysis**		
392-953 (562bp)<br>Bra7 Mt<br>Overlapping PCR	115-Bra7-Mt-F<br>ACAATagaGAAAAGTGGGTTAGCAGGT<br>562-F<br>acgtctcgagCGCAATGCAGCAAGCGATG	953-R<br>acgttctagaTTCCAGCCACCCCTTAAAGT<br>458-Bra7-Mt-R<br>CCCACTTTTCtctATTGTGTTTCTA
392-953 (562bp)<br>Bra6 Mt<br>Overlapping PCR	334-Bra6-Mt-F<br>TCAAGCGAGACGcccaATCAAGTTGCTAAGG<br>562-F<br>acgtctcgagCGCAATGCAGCAAGCGATG	953-R<br>acgttctagaTTCCAGCCACCCCTTAAAGT<br>267-Bra6-Mt-R<br>TAGCAACTTGATtgggCGTCTCGCTTGAGCGG

392-953 (562bp)<br>Bra5+Bra7 Mts<br>Overlapping PCR	196-Bra5-Mtt-F<br>CGTaccAGCTATCTGTGTC<br>562-F<br>acgtctcgagCGCAATGCAGCAAGCGATG	953-R<br>acgttctagaTTCCAGCCACCCCTTAAAGT<br>381-Bra5-Mt-R<br>GACACAGATAGCTggtACG
***Spalt-like* Notochord CRM**		
**1. Identification of the notochord enhancer region**		
UR and Int 1 (1800bp)<br>ATAC-Seq Peak: 1494	1800-F<br>acgtctcgagGCATTGGGTGCATTGCTAAC	1800-R<br>acgttctagaTGATTTACGTCATAATA
UR and Int 1 (933bp)<br>ATAC-Seq Peak: 1494	933-F<br>acgtctcgagCTGCACGATATAATTGTGCG	933-R<br>acgttctagaACTTATTTCGCGTCATTCGC
UR and Int 1 (805bp)<br>ATAC-Seq Peak: 1494	805-F<br>acgtctcgagGCATTGGGTGCATTGCTAAC	805-R<br>acgttctagaCGCACAATTATATCGTGCAG
**2. Truncations**		
UR and Int 1, 1-492 (492bp)	933-F<br>acgtctcgagCTGCACGATATAATTGTGCG	492-R<br>acgttctagaATTAGTTGGGCAGTGGTCG
UR and Int 1, 474-933 (460bp)<br>ATAC-Seq Peak: 1494	460-F<br>acgtctcgagCGACCACTGCCCAACTAAT	933-R<br>acgttctagaACTTATTTCGCGTCATTCGC
UR and Int 1, 474-723 (250bp)<br>ATAC-Seq Peak: 1494	460-F<br>acgtctcgagCGACCACTGCCCAACTAAT	250-R<br>acgttctagaTCTTCCAGCAGAAAAATGACGC
UR and Int 1, 664-933 (270bp)<br>ATAC-Seq Peak: 1494	270-F<br>acgtctcgagGCGGGATCTGCCACCTTTAT	933-R<br>acgttctagaACTTATTTCGCGTCATTCGC
UR and Int 1, 664-814 (151bp)<br>ATAC-Seq Peak: 1494	270-F<br>acgtctcgagGCGGGATCTGCCACCTTTAT	151-F<br>acgttctagaTTCGCGTCATAGAATCTCAACT
UR and Int 1, 757-933 (177bp)<br>ATAC-Seq Peak: 1494	177-F<br>acgtctcgagAATGTTGCAGCCACTGATTAAA	933-R<br>acgttctagaACTTATTTCGCGTCATTCGC
UR and Int 1, 757-916 (160bp)<br>ATAC-Seq Peak: 1494	177-F<br>acgtctcgagAATGTTGCAGCCACTGATTAAA	160-R<br>acgttctagaCGCGACTCACCTTTGGGATA
UR and Int 1, 766-916 (151bp)<br>ATAC-Seq Peak: 1494	151-F<br>acgtctcgagGCCACTGATTAAACAATTGTAACTT	160-R<br>acgttctagaCGCGACTCACCTTTGGGATA
UR and Int 1, 791-916 (126bp)	126-F	160-R

ATAC-Seq Peak: 1494	acgtctcgagCAAGTTGAGATTCTATGACGCGA	acgttctagaCGCGACTCACCTTTGGGATA
**3. Mutation Analysis**		
766-916 (151bp)<br>Fox Mt	151-Fox-Mt-F<br>acgtctcgagGCCACTGATTcccCAATTGTAACTT	160-R<br>acgttctagaCGCGACTCACCTTTGGGATA
766-916 (151bp)<br>AP1+Fox Mt	151-Fox+AP1-Mts-F<br>acgtctcgagGCCACTGATTcccCAATTGTAACTTCAAGTTGAGATTCTATtttGCGA	160-R<br>acgttctagaCGCGACTCACCTTTGGGATA
766-916 (151bp)<br>E-box Mt	151-Ebox-Mt-F<br>acgtctcgagGCCACTGATTAAAagATcaTAACTTcaagt	160-R<br>acgttctagaCGCGACTCACCTTTGGGATA

*Abbreviations: UR, upstream region; Int, intron; HD, homeodomain.

**Table S5. Oligonucleotide primers used for cloning the *C. savignyi* genomic fragments corresponding to *C. robusta* notochord CRMs.**		
**Construct name and size**	**Forward Primer** (5'→3') Restriction site: XhoI (ctcgag)	**Reverse Primer** (5'→3') Restriction site: XbaI (tctaga)
***C. savignyi Aff4* Notochord CRM**		
529bp	529-F	529-R
	atcgctcgagTTCCCATGTATGT TCCAATAGTCTT	atgctctagaTGCTCTAAAAGATG AATTGACGC
***C. savignyi CasZ1* Notochord CRM**		
4755bp	475-F	475-R
	atcgctcgagTATAATCTGGGA TCATTATACGGCT	atgctctagaATACAAACTTCTTCA GCAAAACCT
***C. savignyi Cnot11* Notochord CRM**		
665bp	665-F	665-R
	atcgctcgagTGGATAAATCTC GCTTAAATCAGTG	atgctctagaAGTTTTCCAGGGGG AATTCTTT
***C. savignyi Etv1* Notochord CRM**		
567bp	567-F atcgctcgagAACCCGCAGATT ACGGTTATGTA	567-R atgctctagaTCGATTTTCCCAGTG CTAGGC
***C. savignyi Fli/Erg* Notochord CRM**		
576bp	576-F atcgctcgagACTGCAGTATCA TCCAAACAGC	576-R atgctctagaCAAAATGGTCCACT TGTGCCT
***C. savignyi Islet1* Notochord CRM**		
52bbp	522-F atcgctcgagGACGATCCGGCA CCTGTAAA	522-R atgctctagaGCGAGTTTCAGAAC TGCCAA
***C. savignyi Lmx1-r* Notochord CRM**		
469bp	469-F atcgctcgagAGCACCATTGTG TAACGGCT	469-R atgctctagaTCAGTGAAACTATT ATTGCCGATCC
***C. savignyi Mxd1* Notochord CRM**		
702bp	702-F atcgctcgagTGACGACGGGCG TTAAAACA	702-R atgctctagaAGCATGGATATGCG ACACCT
***C. savignyi Ror-a* Notochord CRM**		
542bp	542-F atcgctcgagCAGGATGCTTCC CGCACTG	542-R atgctctagaATGGCGGGCAATAA GTACCG

C. savignyi Spalt-like **Notochord CRM**		
421bp	421-F<br>atcgctcgagCCTTTTTCCCTTCTTCTTTCTCG	421-R<br>atgctctagaTGGGCCGTTTTCTCAATCTT

Supplemental References

133. Satou, Y., Kawashima, T., Shoguchi, E., Nakayama, A. & Satoh, N. An integrated database of the ascidian, Ciona intestinalis: towards functional genomics. *Zoological science* **22**, 837-843 (2005).

135. Satou, Y. *et al.* Improved genome assembly and evidence-based global gene model set for the chordate Ciona intestinalis: new insight into intron and operon populations. *Genome Biology* **9**, R152, doi:10.1186/gb-2008-9-10-r152 (2008).
