# Supplementary material for: Cis-regulatory interfaces reveal the molecular mechanisms underlying the notochord gene regulatory network of Ciona
Source: Nat Commun. 2024 Apr 8;15:3025. doi: 10.1038/s41467-024-46850-3 (PMC11001920; doi:10.1038/s41467-024-46850-3)
Supplement: Supplementary file 3 — Reporting Summary [file 41467_2024_46850_MOESM3_ESM.pdf]

nature portfolio

Corresponding author(s): Anna Di Gregorio

Last updated by author(s): Feb 15, 2024

Reporting Summary

Nature Portfolio wishes to improve the reproducibility of the work that we publish. This form provides structure for consistency and transparency in reporting. For further information on Nature Portfolio policies, see our Editorial Policies and the Editorial Policy Checklist.

Statistics

For all statistical analyses, confirm that the following items are present in the figure legend, table legend, main text, or Methods section.

n/a	Confirmed	
☐	☒	The exact sample size (*n*) for each experimental group/condition, given as a discrete number and unit of measurement
☐	☒	A statement on whether measurements were taken from distinct samples or whether the same sample was measured repeatedly
☐	☒	The statistical test(s) used AND whether they are one- or two-sided<br>*Only common tests should be described solely by name; describe more complex techniques in the Methods section.*
☒	☐	A description of all covariates tested
☒	☐	A description of any assumptions or corrections, such as tests of normality and adjustment for multiple comparisons
☐	☒	A full description of the statistical parameters including central tendency (e.g. means) or other basic estimates (e.g. regression coefficient) AND variation (e.g. standard deviation) or associated estimates of uncertainty (e.g. confidence intervals)
☐	☒	For null hypothesis testing, the test statistic (e.g. *F*, *t*, *r*) with confidence intervals, effect sizes, degrees of freedom and *P* value noted<br>*Give P values as exact values whenever suitable.*
☒	☐	For Bayesian analysis, information on the choice of priors and Markov chain Monte Carlo settings
☒	☐	For hierarchical and complex designs, identification of the appropriate level for tests and full reporting of outcomes
☒	☐	Estimates of effect sizes (e.g. Cohen's *d*, Pearson's *r*), indicating how they were calculated

Our web collection on statistics for biologists contains articles on many of the points above.

Software and code

Policy information about availability of computer code

Data collection	Image data were collected using either a Leica DMi8 SP8 confocal microscope or a Leica DMR microscope connected to a Leica DC500 digital camera.
Data analysis	Fluorescence confocal images were processed using the Fiji Open Source Software (Version: 2.14.0/1.54f).

For manuscripts utilizing custom algorithms or software that are central to the research but not yet described in published literature, software must be made available to editors and reviewers. We strongly encourage code deposition in a community repository (e.g. GitHub). See the Nature Portfolio guidelines for submitting code & software for further information.

Data

Policy information about availability of data

All manuscripts must include a data availability statement. This statement should provide the following information, where applicable:

- Accession codes, unique identifiers, or web links for publicly available datasets
- A description of any restrictions on data availability
- For clinical datasets or third party data, please ensure that the statement adheres to our policy

All data generated during this study are included in this published article and its supplementary information files. Source data are provided with this paper. Data sharing is not applicable to this article as no datasets were generated during the current study.

Research involving human participants, their data, or biological material

Policy information about studies with human participants or human data. See also policy information about sex, gender (identity/presentation), and sexual orientation and race, ethnicity and racism.

Reporting on sex and gender	N/A
Reporting on race, ethnicity, or other socially relevant groupings	N/A
Population characteristics	N/A
Recruitment	N/A
Ethics oversight	N/A

Note that full information on the approval of the study protocol must also be provided in the manuscript.

Field-specific reporting

Please select the one below that is the best fit for your research. If you are not sure, read the appropriate sections before making your selection.

[x] Life sciences [ ] Behavioural & social sciences [ ] Ecological, evolutionary & environmental sciences

For a reference copy of the document with all sections, see nature.com/documents/nr-reporting-summary-flat.pdf

Life sciences study design

All studies must disclose on these points even when the disclosure is negative.

Sample size	The sample size was determined on the basis of previously published research.
Data exclusions	No data relevant to this project were excluded from the analysis.
Replication	Each transgenic experiment was carried out at least 3 times in the same conditions, using animals collected on different dates from the same geographic location. Whenever an attempt to replication failed, due to the conditions/fertility/development of the animals, or whenever the replication was successful but did not yield a sufficient number of embryos, the experiment was repeated on a different batch of animals.
Randomization	N/A
Blinding	N/A

Reporting for specific materials, systems and methods

We require information from authors about some types of materials, experimental systems and methods used in many studies. Here, indicate whether each material, system or method listed is relevant to your study. If you are not sure if a list item applies to your research, read the appropriate section before selecting a response.

Materials & experimental systems

n/a	Involved in the study	
[ ]	[x]	Antibodies
[x]	[ ]	Eukaryotic cell lines
[x]	[ ]	Palaeontology and archaeology
[ ]	[x]	Animals and other organisms
[x]	[ ]	Clinical data
[x]	[ ]	Dual use research of concern
[x]	[ ]	Plants

Methods

n/a	Involved in the study	
[x]	[ ]	ChIP-seq
[x]	[ ]	Flow cytometry
[x]	[ ]	MRI-based neuroimaging

Antibodies

Antibodies used	A specific polyclonal antibody raised in rabbit against Ciona Brachyury, designed and validated in our lab, and commercially available antibodies against Beta-galactosidase and Cas9.
Validation	Validation of the in-house anti-Ciona-Brachyury antibody is reported in Aihara et al., Mar Biotechnol (NY). 2013 Oct;15(5):520-5. doi: 10.1007/s10126-013-9504-5. PMID: 23592257; PMCID: PMC3744622. Dilution: 1:250.

Anti-beta-galactosidase: mouse beta-galactosidase (cat. number: A-11132; Promega, Madison, WI), 1:500 dilution.
Cas9 antibody 4G10 (cat. number: C15200216-10; Diagenode, Denville, NJ), 1:500 dilution.
Goat anti-rabbit Alexa Fluor 488 (cat. number: A27034; Invitrogen, Waltham, MA), 1:1000 dilution.
Goat anti-mouse Alexa Fluor 555 (cat. number: A21422; Invitrogen, Waltham, MA), 1:1000 dilution.

Animals and other research organisms

Policy information about studies involving animals; ARRIVE guidelines recommended for reporting animal research, and Sex and Gender in Research

Laboratory animals	This study did not involve laboratory animals.
Wild animals	Adult Ciona robusta (formerly Ciona intestinalis type A; Pennati et al., 2015) wild animals were collected by M-REP (Carlsbad, CA, USA) in marinas located in the vicinity of San Diego (CA, USA).
Reporting on sex	Ciona robusta are hermaphrodites.
Field-collected samples	Adult Ciona robusta (formerly Ciona intestinalis type A; Pennati et al., 2015) collected from marinas located in the vicinity of San Diego (CA, USA) were purchased from M-REP (Carlsbad, CA, USA) and kept at 19°C in an aquarium with refrigerated recirculating artificial seawater. Gametes were removed from the gonoducts in order to carry out in vitro fertilization, and after that the dead animals were disposed of.
Ethics oversight	N/A

Note that full information on the approval of the study protocol must also be provided in the manuscript.

Plants

Seed stocks	N/A
Novel plant genotypes	N/A
Authentication	N/A
